# Salbutamol repurposing ameliorates neuromuscular junction defects and muscle atrophy in *Col6a1^−/−^* mouse model of collagen VI‐related myopathies

**DOI:** 10.1002/ctm2.1688

**Published:** 2024-07-10

**Authors:** Sonia Calabrò, Leonardo Nogara, Yongzhi Jian, Manuel Valentin, Dario Bizzotto, Paola Braghetta, Loris Russo, Lisa Gambarotto, Bert Blaauw, Said Hashemolhosseini, Paolo Bonaldo, Matilde Cescon

**Affiliations:** ^1^ Department of Molecular Medicine University of Padova Padova Italy; ^2^ Department of Biology University of Padova Padova Italy; ^3^ Department of Biomedical Sciences University of Padova Padova Italy; ^4^ Venetian Institute of Molecular Medicine Padova Italy; ^5^ Department of Pharmaceutical and Pharmacological Sciences University of Padova Padova Italy; ^6^ Institut für Biochemie Friedrich‐Alexander‐Universität Erlangen‐Nürnberg Erlangen Germany

1

Dear Editor,

Collagen VI (ColVI)‐related myopathies are a distinct group of progressive muscle disorders, which include Ullrich congenital muscular dystrophy (UCMD) and Bethlem myopathy (BM), for which no therapy is yet available. In the last two decades, much effort was spent on the elucidation of the pathogenic mechanisms underlying ColVI‐related myopathies, also by taking advantage of the ColVI null (*Col6a1* ^−/−^) mouse model,^1^ in order to identify druggable targets for prospective therapies.^2^, ^3^, ^4^

The results obtained within this work provide a proof‐of‐concept for the repurposing of the Food and Drug Administration (FDA)‐approved salbutamol in the context of ColVI‐related myopathies, as they demonstrate that systemic salbutamol administration can recover the major structural and functional neuromuscular junction (NMJ) defects occurring in murine muscles downstream ColVI deficiency, and previously described also in patients.^5^

We selected salbutamol for three main reasons: (i) it was previously reported to ameliorate NMJ structure and electrophysiological cues in animal models of human NMJ‐related disorders; (ii) it is an FDA‐approved drug already used with success as an off‐label medication in several diseases displaying not only primary but also secondary NMJ defects, including Pompe disease, spinal muscular atrophy and Duchenne, Becker and facioscapulohumeral muscular dystrophies; (iii) it was proven to be safe in the majority of the open clinical trials and therefore eligible for future rapid translational applications in the clinical settings.^6^

Five‐month‐old wild type (WT) and *Col6a1* ^−/−^ male mice were treated with either vehicle or salbutamol at two different doses, 4 and 8 mg/kg/die, subcutaneously administered via Alzet osmotic minipumps for 28 days (Figure S1 and Supporting Information).

First, we investigated the impact of salbutamol on NMJ structural defects and found that the abnormally increased NMJ fragmentation displayed by vehicle‐treated *Col6a1^−/−^* diaphragms, compared to WT animals (Figure 1A–C), was recovered by treatment with salbutamol at the higher dose (Figure 1D–F). Furthermore, morphometric analysis of NMJs showed that both low‐ and high‐dose salbutamol induced a significant increase in postsynaptic terminal areas and compactness in *Col6a1^−/−^* mice (Figures 1G–L and S2A), coherently with reports of salbutamol beneficial effects in expanding NMJ size in animal models of congenital myasthenic syndromes (CMS).^7^, ^8^ The analyses performed on treated WT mice confirmed the general trend of salbutamol in increasing NMJ size (Figure S2B–H). To evaluate whether salbutamol also ameliorated neuromuscular transmission, we first monitored the four‐limb hanging test performance. Besides confirming a marked difference between WT and *Col6a1^−/−^* mice (Figure 1M), the test highlighted an improved performance of *Col6a1^−/−^* mice treated with high‐dose salbutamol (Figure 1N,O). Moreover, ex vivo electrophysiological recordings performed in diaphragm muscles revealed that high‐dose salbutamol treatment recovered the NMJ functional parameters found to be altered in *Col6a1^−/−^* mice, when compared to WT animals, including a lower amplitude of nerve‐evoked endplate potentials and of miniature endplate potentials (mEPP), a reduced quantal content and input resistance, as well as decreased endplate currents, miniature endplate currents and mEPP frequency (Figure 1P–V). Notably, assessment of neuromuscular transmission by repetitive stimulation of the phrenic nerve for 25 pulses at 5 Hz further highlighted that the higher decrement registered upon vehicle treatment in *Col6a1^−/−^* mice, when compared to WT animals, was also rescued by salbutamol (Figure 1W), clearly indicating that high‐dose salbutamol treatment not only ameliorates NMJ fragmentation but also elicits NMJ functional improvement in *Col6a1^−/−^* mice.

**FIGURE 1:**
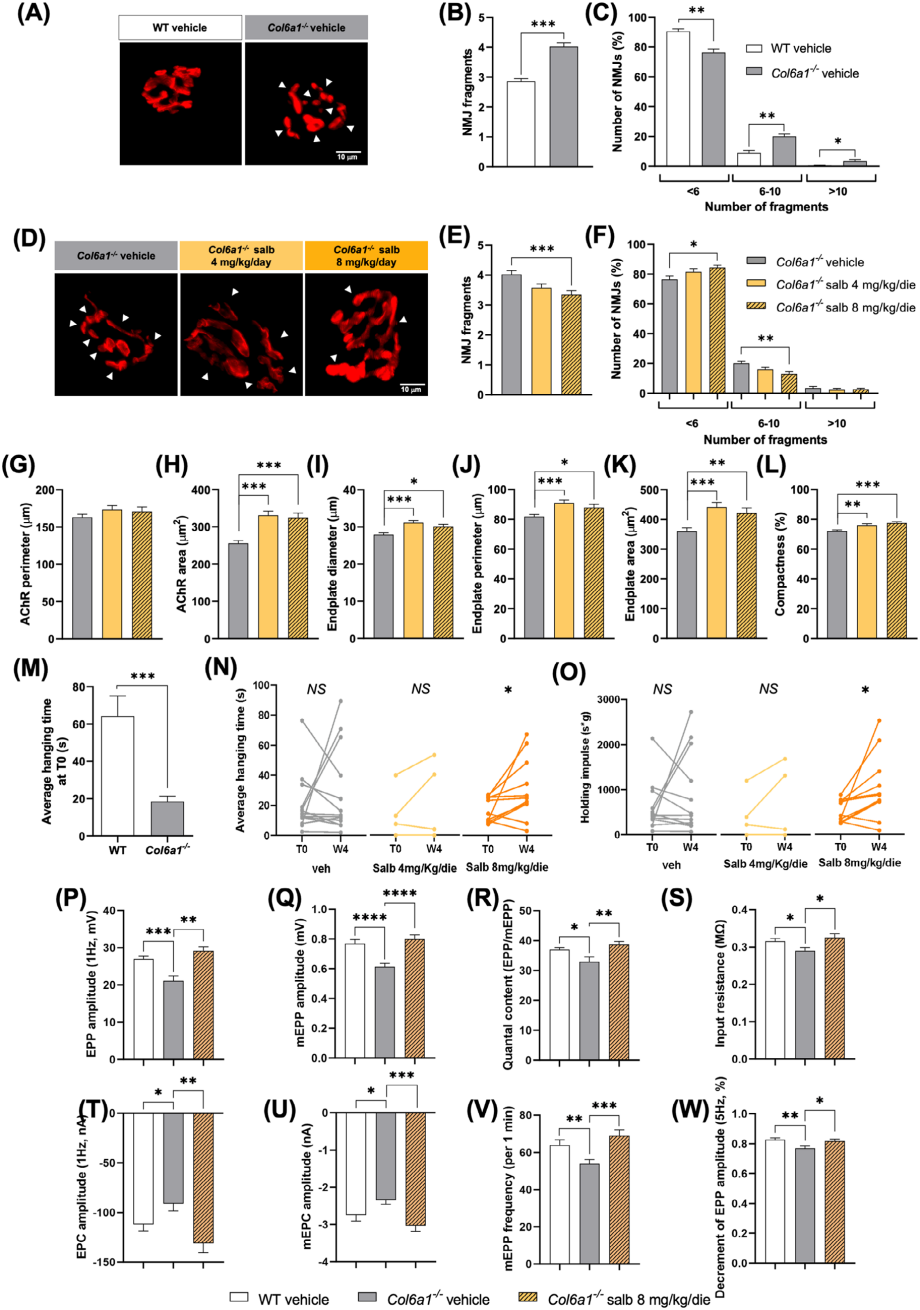
Salbutamol treatment ameliorates neuromuscular junction (NMJ) fragmentation, promotes NMJ remodelling and induces NMJ functional improvement in *Col6a1^−/−^* mice. (A) Whole‐mount hemidiaphragms preparations of vehicle‐treated WT and *Col6a1^−^* ^/−^ mice, stained with 555‐BTX. White arrowheads indicate AChR cluster fragments. Scale bar, 10 µm. (B) Quantification of the average number of fragments per NMJ in vehicle‐treated WT versus *Col6a1^−^* ^/−^ mice. Data are shown as mean ± s.e.m. (***p < .001; Mann–Whitney test; *n *= 546–604 NMJs from 5 to 7 mice, each group). (C) Percentage of total NMJs displaying the number of fragments reported in the *x*‐axis in vehicle‐treated WT versus *Col6a1^−^* ^/−^ mice. Data are shown as mean ± s.e.m. (*p < .05; **p < .01; Mann–Whitney test; *n *= 5–7 mice, each group). (D) Whole‐mount hemidiaphragm preparations of vehicle‐ and salbutamol‐treated *Col6a1^−^* ^/−^ mice, stained with 555‐BTX to highlight AChR cluster fragmentation (white arrowheads). Scale bar, 10 µm. (E) Quantification of the average number of fragments per NMJ in vehicle‐ versus salbutamol‐treated *Col6a1^−^* ^/−^ mice. Data are shown as mean ± s.e.m. (***p < .001; Kruskal–Wallis test with Dunn's post hoc test for multiple comparisons; *n *= 352–604 NMJs from 4 to 7 mice, each group). (F) Percentage of total NMJs displaying the number of fragments reported in the *x*‐axis in vehicle‐ versus salbutamol‐treated *Col6a1^−^* ^/−^ mice. Data are shown as mean ± s.e.m. (*p < .05; **p < .01; Kruskal–Wallis test with Dunn's post hoc test for multiple comparisons; *n *= 4–7 mice, each group). (G–L) Quantitative analysis of postsynaptic parameters in diaphragm muscles of vehicle‐ and salbutamol‐treated *Col6a1^−^* ^/−^ mice, showing AChR perimeter (G) and area (H), and endplate diameter (I), perimeter (J), area (K) and compactness (L). Data are shown as mean ± s.e.m. (*p < .05, **p < .01; ***p < .001; Kruskal–Wallis test with Dunn's post hoc test for multiple comparison; *n *= 96–147 NMJs from 4 to 7 mice, each group). (M) Average hanging time of WT and *Col6a1^−^* ^/−^ mice subjected to the four‐limb hanging test at T0 (before treatment). Data are shown as mean ± s.e.m. (***p < .001; Mann–Whitney test; *n *= 28–30 mice, each group). (N, O) Individual four‐limb hanging performances of *Col6a1^−^* ^/−^ mice before (T0) and after (W4) 4‐week‐long treatment with vehicle or salbutamol. The average hanging time (N) and the holding impulse (O) are shown as outcome measures (*p < .05; *NS*, not significant; two‐tailed paired *t*‐test for parametric data and Wilcoxon matched pairs signed rank test for non‐parametric data; *n *= 5–15 mice, each group). (P–W) Quantitative analysis of electrophysiological parameters measured in ex vivo diaphragm preparations of vehicle‐treated WT mice, vehicle‐treated *Col6a1^−^* ^/−^ mice and 8 mg/kg/die salbutamol‐treated *Col6a1^−^* ^/−^ mice, showing nerve‐evoked endplate potentials (EPP) (P) and miniature endplate potentials (mEPP) (Q) amplitudes, quantal content (R), input resistance (S), endplate currents (EPC) (T), miniature endplate currents (mEPC) (U), frequency of mEPP (V), and EPP amplitude decrement at 5 Hz (W). Error bars indicate s.e.m. (*p < .05; **p < .01; ***p < .001; unpaired two‐tailed Student's *t*‐test; *n *= 8 mice, each group). AChR, acetylcholine receptor; Salb, salbutamol; veh, vehicle; WT, wild type.

Since β2‐agonists are well‐known anabolic agents,^9^ we hypothesised that *Col6a1^−/−^* muscles could benefit per se from salbutamol treatment. Thus, we evaluated salbutamol impact on muscle mass, attesting a significant increase of body weight and of the absolute and normalised tibialis anterior (TA) and gastrocnemius muscle weight both in *Col6a1^−/−^* and WT mice (Figures 2A–C and S3A–F). Consistently, morphometric analysis of TA cross‐sections revealed a significant increase in average cross‐sectional area and minimum Feret's diameter elicited by high‐dose salbutamol treatment in *Col6a1^−/−^* mice (Figure 2E,F), paralleled by a shift of myofibre size distribution towards larger myofibre classes (Figure 2G–L) and by a reduction in myofibre density (Figure 2M). A similar shift in fibre size was detectable in salbutamol‐treated WT muscles (Figure S3G–O), whereas myofibre density was not significantly affected (Figure S3P). Interestingly, while salbutamol treatment in either *Col6a1^−/−^* and WT mice did not influence the occurrence of centronucleated fibres (Figures 2N and S3Q), a higher proportion of regenerating fibres, together with increased *Myh3* transcript levels, was detectable in muscles of both genotypes upon high‐dose treatment (Figures 2O–Q and S3R–T). Such results validated the concept that salbutamol not only promotes muscle remodelling in WT animals but also counteracts muscle mass loss and promotes muscle regeneration in *Col6a1^−/−^* mice.

**FIGURE 2:**
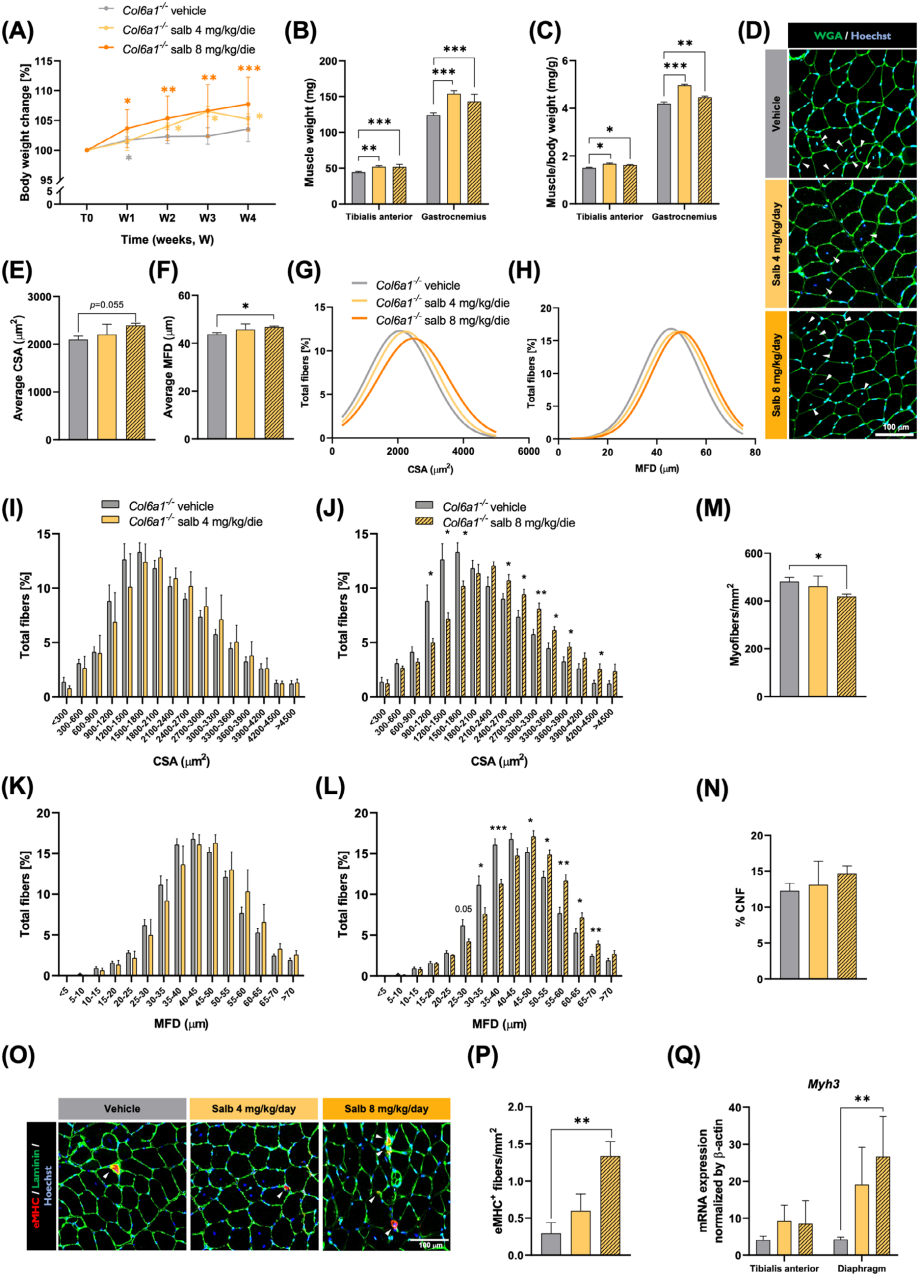
Salbutamol remodels skeletal muscle in *Col6a1^−^* ^/−^ mice. (A) Growth curve showing changes in body weight over the 4 weeks of treatment (W1–W4) in vehicle‐ and salbutamol‐treated *Col6a1* ^−/−^ mice. The values registered at each time point are expressed as a percentage of the value measured before treatment (T0). Data are shown as mean ± s.e.m. (*p < .05; **p < .01; ***p < .001, compared to T0 within the same group; 2‐way analysis of variance (ANOVA) test with Dunnet's post hoc test for multiple comparisons; *n *= 4–13 mice, each group). (B, C) Absolute (B) and normalised (C) weight of tibialis anterior (TA) and gastrocnemius muscles of vehicle‐ and salbutamol‐treated *Col6a1^−/‐^* mice. Data are shown as mean ± s.e.m. (*p < .05; **p < .01; ***p < .001; Kruskal–Wallis test with Dunn's post hoc test for multiple comparisons for non‐parametric data or one‐way ANOVA test with Dunnett's post hoc test for multiple comparisons for parametric data; *n *= 8–32 muscles, each group). (D) Representative fluorescence microscopy images of cross‐sections of TA muscle from vehicle‐ and salbutamol‐treated *Col6a1^−/‐^* mice, stained with fluorophore‐conjugated wheat germ agglutinin (WGA, green) and Hoechst (blue). Arrowheads point at centrally located nuclei. Scale bar, 100 µm. (E, F) Quantification of myofibre cross‐sectional area (CSA) (E) and minimum Feret's diameter (MFD) (F) in vehicle‐ and salbutamol‐treated *Col6a1^−/−^* mice. Data are shown as mean ± s.e.m. (*p < .05; one‐way ANOVA test with Dunnett's post hoc test for multiple comparisons; *n *= 3–8 mice, each group). (G, H) Graphical representation of CSA (G) and MFD (H) distribution among myofibres. The curves were fitted to data using non‐linear regression (Gaussian). (I–L) Comparison of CSA (I, J) and MFD (K, L) distribution among myofibres between vehicle‐treated and low‐dose (I, K) or high‐dose (J, L) salbutamol‐treated *Col6a1^−/−^* muscles. Data are shown as mean ± s.e.m. (*p < .05; **p < .01; ***p < .001; multiple unpaired two‐tailed Student's *t*‐tests; *n *= 3–8 mice, each group). (M) Quantification of myofibre density, calculated as the number of myofibres per total area, in vehicle‐ and salbutamol‐treated *Col6a1^−/−^* mice. Data are shown as mean ± s.e.m. (*p < .05; one‐way ANOVA test with Dunnett's post hoc test for multiple comparisons; *n *= 3–8 mice, each group). (N) Quantification of the percentage of centrally nucleated fibres (CNF) per muscle section in vehicle‐ and salbutamol‐treated *Col6a1^−/−^* mice. Data are shown as mean ± s.e.m. (one‐way ANOVA with Dunnet's post hoc test for multiple comparisons; *n *= 3–8 mice, each group). (O) Representative confocal micrographs of TA cross‐sections from vehicle‐ and salbutamol‐treated *Col6a1^−/−^* mice, stained with Hoechst (blue) and antibodies for embryonic myosin heavy chain (eMHC, red) and laminin (green). White arrowheads indicate eMHC‐positive myofibres. Scale bar, 100 µm. (P) Quantification of eMHC‐positive myofibres per area unit (in mm^2^), based on confocal micrographs as in (O). Data are shown as mean ± s.e.m. (**p < .01; one‐way ANOVA test with Dunnett's post hoc test for multiple comparisons; *n* = 3–6 mice, each group). (Q) Real‐time quantitative polymerase chain reaction (RT‐qPCR) analysis of myosin heavy chain 3 (Myh3) transcript levels in TA and diaphragm muscles of vehicle‐ and salbutamol‐treated *Col6a1^−/−^* mice. Data are shown as mean ± s.e.m. (**p < .01; one‐way ANOVA test with Dunnett's post hoc test for multiple comparisons; *n* = 4–9 mice, each group). Salb, salbutamol.

In keeping with literature, showing that β2‐agonists can induce slow‐oxidative to fast‐glycolytic myofibre switch in rodents,^9^ despite the absence of any overt change in succinate dehydrogenase staining in WT and *Col6a1^−/−^* muscles (Figures 3A,B and S4A,B), an upregulation of *Myh4* gene (Figures 3C,D and S4C,D) and an increased percentage of type IIB fibres (Figure 3E,F) were detected upon salbutamol treatment in *Col6a1^−/−^* mice, compared to the corresponding vehicle‐treated mice. Similar changes were not detectable in salbutamol‐treated WT muscles (Figure S4E,F).

**FIGURE 3:**
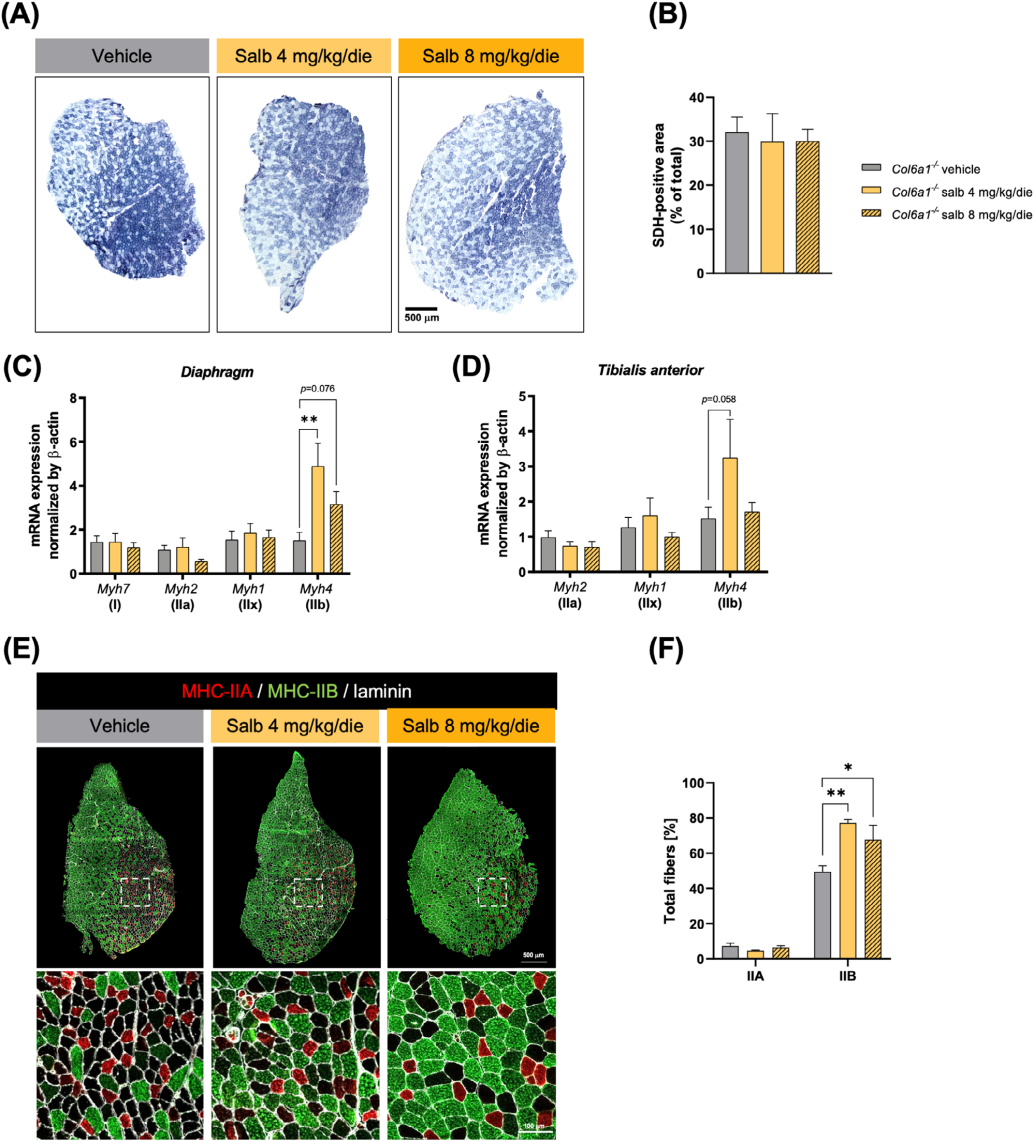
Salbutamol modifies muscle fibre composition in *Col6a1* ^−^ *^/^* ^−^ mice. (A) Representative reconstruction of optical micrographs of TA cross‐sections from vehicle‐ and salbutamol‐treated *Col6a1^−/^* ^−^ mice, following histochemical succinate dehydrogenase (SDH) staining. SDH‐positive fibres are stained in dark blue, whereas SDH‐negative fibres are stained in light blue. Scale bar, 500 µm. (B) Quantification of SDH‐positive area, determined by SDH staining as in (A). Data are shown as mean ± s.e.m. (Kruskal–Wallis test with Dunn's post hoc test for multiple comparisons; *n *= 3–6 mice, each group). (C, D) RT‐qPCR quantification of the levels of transcripts for different myosin heavy chain (MHC) isoforms in the diaphragm (C) and TA (D) muscles of vehicle‐ and salbutamol‐treated *Col6a1^−/‐^* mice. Data are shown as mean ± s.e.m. (**p < .01; one‐way ANOVA test with Dunnett's post hoc test for multiple comparisons; *n *= 4–9 mice, each group). (E) Representative merged fluorescence microscopy images of whole TA cross‐sections from vehicle‐ and salbutamol‐treated *Col6a1^−/−^* mice, stained with antibodies against MHC‐IIA (red), MHC‐IIB (green) and laminin (white). Dotted white squares highlight the areas shown at higher magnification in the bottom panels. Scale bars, 500 µm (top panels) or 100 µm (bottom panels). (F) Quantification of the percentages of type IIA and type IIB fibres, based on immunofluorescent images as in (E). Data are shown as mean ± s.e.m. (*p < .05; **p < .01; one‐way ANOVA test with Dunnett's post hoc test for multiple comparisons; *n *= 3–5 mice, each group). Salb, salbutamol.

We then evaluated salbutamol's ability to increase muscle strength, by performing in vivo tetanic force measurements on gastrocnemius muscles of WT and *Col6a1^−/−^* mice, following either salbutamol or vehicle administration. The impaired muscle strength of *Col6a1^−/−^*, compared to WT mice, both in terms of absolute and normalised force (Figure 4A,B), resulted ameliorated by salbutamol since it induced a significant gain in the absolute force, mostly evident at intermediate stimulation frequencies (Figure 4C), which, notably, remained significant even when normalised to muscle weight upon high‐dose treatment (Figure 4D). In WT mice, treatments determined a significant gain only in absolute force at intermediate frequencies of stimulation (Figure S5A,B). Of note, the curves corresponding to the normalised force of vehicle‐treated WT mice displayed a complete overlap with those of salbutamol‐treated *Col6a1^−/−^* mice at intermediate stimulation frequencies (Figure S5C,D), demonstrating that salbutamol induces a substantial amelioration of in vivo muscle strength.

**FIGURE 4:**
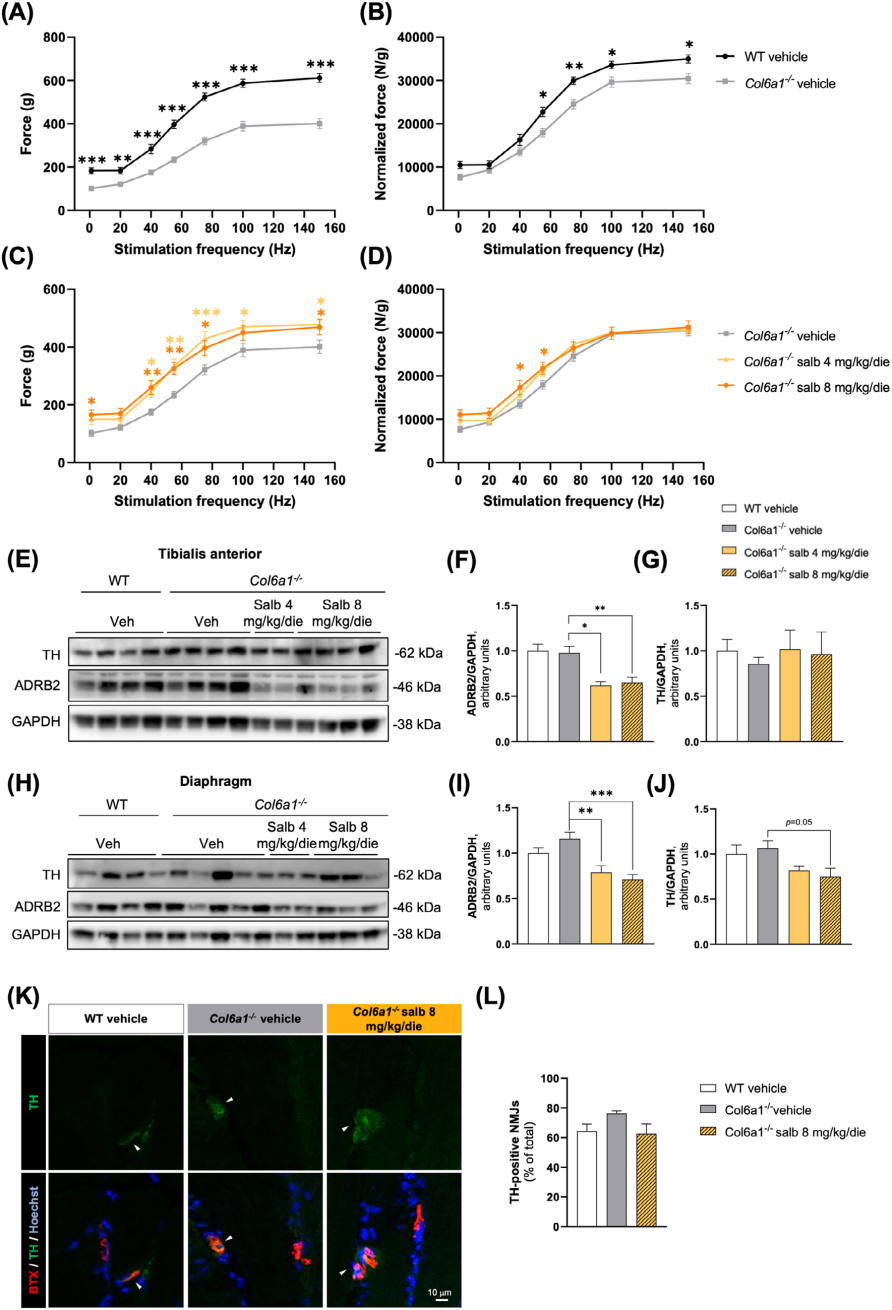
One‐month salbutamol treatment ameliorates muscle force in *Col6a1^−/−^* mice and leads to ADRB2 downregulation. (A, B) Absolute (A) and normalised (B) in vivo force measurements of gastrocnemius muscle from vehicle‐treated WT and *Col6a1^−^* ^/^ *^−^* mice. Whole curves of stepwise stimulation frequencies are shown. Tetanus was reached at a stimulation frequency of 100 Hz. Data are shown as mean ± s.e.m. (*p < .05; **p < .01; ***p < .001, comparing vehicle treatment in different genotypes at each stimulation frequency; multiple unpaired *t*‐tests with Holm–Sidak method to correct for multiple comparisons; *n *= 12–16 limbs, each group). (C, D) Absolute (C) and normalised (D) in vivo force measurements of gastrocnemius muscle from vehicle‐treated and salbutamol‐treated *Col6a1^−^* ^/^ *^−^* mice. Whole curves of stepwise stimulation frequencies are shown. Tetanus was reached at a stimulation frequency of 100 Hz. Data are shown as mean ± s.e.m. (*p < .05; **p < .01; ***p < .001, comparing salbutamol treatment to vehicle treatment within the same genotype, at each stimulation frequency; two‐way ANOVA with Dunnett's post hoc test for pairwise comparisons; *n *= 8–12 limbs, each group). (E) Representative western blot for ADRB2 and TH in total protein extracts of TA muscle from vehicle‐treated WT mice and vehicle‐ and salbutamol‐treated *Col6a1^−/−^* mice. Glyceraldehyde‐3‐phosphate dehydrogenase (GAPDH) was used as a loading control. (F) Densitometric quantification of ADRB2 levels normalised to GAPDH levels, determined by three independent western blot experiments as in (E). Data are shown as mean ± s.e.m. (*p < .05; **p < .01; one‐way ANOVA with Dunnett's post hoc test for multiple comparisons; *n* = 4–9 mice, each group). (G) Densitometric quantification of TH levels normalised to GAPDH levels, determined by two independent western blot experiments as in (E). Data are shown as mean ± s.e.m. (one‐way ANOVA with Dunnett's post hoc test for multiple comparisons; *n *= 4–9 mice, each group). (H) Representative western blot for ADRB2 and TH in total protein extracts of diaphragm muscle from vehicle‐treated WT mice and vehicle‐ and salbutamol‐treated *Col6a1^−^* ^/‐^ mice. GAPDH was used as a loading control. (I) Densitometric quantification of ADRB2 levels normalised to GAPDH levels, determined by two independent western blot experiments as in (G). Data are shown as mean ± s.e.m. (**p < .01; one‐way ANOVA with Dunnett's post hoc test for multiple comparisons; *n *= 4–9 mice, each group). (J) Densitometric quantification of TH levels normalised to GAPDH levels, determined by two independent western blot experiments as in (G). Data are shown as mean ± s.e.m. (one‐way ANOVA with Dunnett's post hoc test for multiple comparisons; *n *= 4–9 mice, each group). (K) Representative fluorescence microscopy images of longitudinal sections of gastrocnemius muscles from vehicle‐treated WT and vehicle‐ and high‐dose salbutamol‐treated *Col6a1^−/−^* mice, stained with an antibody against TH (green), 555‐BTX (red) and Hoechst. Scale bar, 10 µm. (L) Quantification of the percentages of NMJs showing TH staining according to (K). Data are shown as mean ± s.e.m. (Kruskal–Wallis test with Dunn's post hoc test for multiple comparisons; *n *= 3 mice, each group). More than 50 NMJs were analyzed for each sample. ADRB2, adrenergic receptor β2; Salb, salbutamol; TH, tyrosine hydroxylase; Veh, vehicle; WT, wild type.

Finally, β2‐adrenergic receptor protein levels were significantly reduced in both TA and diaphragm muscles of *Col6a1^−/−^* mice after the 4‐week salbutamol treatment at both doses (Figure 4E,F,H,I), consistently with receptor desensitisation^9^ and with a potential undesired mitigation of the biological response to the treatment.

Considering that salbutamol is a sympathomimetic drug and supposing it might reconstitute a defective sympathetic innervation of NMJs of *Col6a1^−/−^* mice, we monitored tyrosine hydroxylase (TH) as a marker for sympathetic neurons. TH protein levels appeared only mildly affected in TA muscles (Figure 4E,G), while in diaphragms, a decrease in TH levels was detectable in high‐dose salbutamol‐treated *Col6a1^−/−^* mice, when compared to vehicle‐treated ones (Figure 4H,J). In situ quantification of NMJs presenting sympathomimetic innervation did not highlight any further difference between vehicle‐treated WT and vehicle‐ or high‐dose salbutamol‐treated *Col6a1^−/−^* mice (Figure 4K,L), and norepinephrine concentration in serum was not affected as well by the different treatments (Figure S6). Therefore, we cannot infer that salbutamol efficacy relies on an overtly impaired sympathetic innervation in *Col6a1^−/−^* mice.

In conclusion, our results show that 1‐month systemic treatment with salbutamol stabilises NMJ structures while ameliorating the myopathic phenotype of *Col6a1^−/−^* mice. By limiting muscle wasting and improving neuromuscular transmission in mice, salbutamol administration may provide clinical benefits for patients. Of note, salbutamol doses adopted here are comparable to those proven to be successful in counteracting CMS phenotype in murine models.^8^ On the other hand, a direct conversion to human application, where the highest effective dosages used in clinics for CMS adult patients range between 6 and 12 mg per die,^10^ is not realistic. In addition, subcutaneous infusion for salbutamol administration is less frequently opted in patients, where the per os route is preferred in the context of CMS and other dystrophies.^6^ Nonetheless, being salbutamol already approved by the FDA, optimised salbutamol‐based therapeutic strategies might be more rapidly translated into clinical trials, an aspect of high relevance in the clinical setting for BM/UCMD patients.

## AUTHOR CONTRIBUTIONS

Matilde Cescon designed the study, acquired funding and supervised analysis; Sonia Calabrò and Matilde Cescon performed in vivo treatments; Sonia Calabrò performed in vivo and ex‐vivo analysis; Leonardo Nogara performed in vivo muscle force measurements; Yongzhi Jian and Manuel Valentin performed ex‐vivo electrophysiological analysis; Dario Bizzotto and Paola Braghetta managed mouse colonies; Loris Russo and Lisa Gambarotto contributed to the analysis; Bert Blaauw and Said Hashemolhosseini supervised in vivo and ex‐vivo electrophysiological analysis, respectively; Paolo Bonaldo provided *Col6a1^−/−^* mice and acquired funding; Sonia Calabrò and Matilde Cescon wrote the original draft; all the other authors contributed to revision of the manuscript.

## CONFLICT OF INTEREST STATEMENT

The authors declare no conflicts of interest.

## ETHICS STATEMENT

Animal procedures were approved by the Animal Ethics Committee of the University of Padova and authorised by the Italian Ministry of Health (Project No. 98/2020‐PR).

## Supporting information

Supporting Information

Supporting Information

## Data Availability

All data needed to evaluate the conclusions in the paper are present in the paper and the Supporting Information.
