# Supplementary material for: Salbutamol repurposing ameliorates neuromuscular junction defects and muscle atrophy in Col6a1−/− mouse model of collagen VI‐related myopathies
Source: Clin Transl Med. 2024 Jul 10;14(7):e1688. doi: 10.1002/ctm2.1688 (PMC11234414; doi:10.1002/ctm2.1688)
Supplement: Supplementary file 1 — Supporting Information [file CTM2-14-e1688-s002.pptx]

## Slide 1
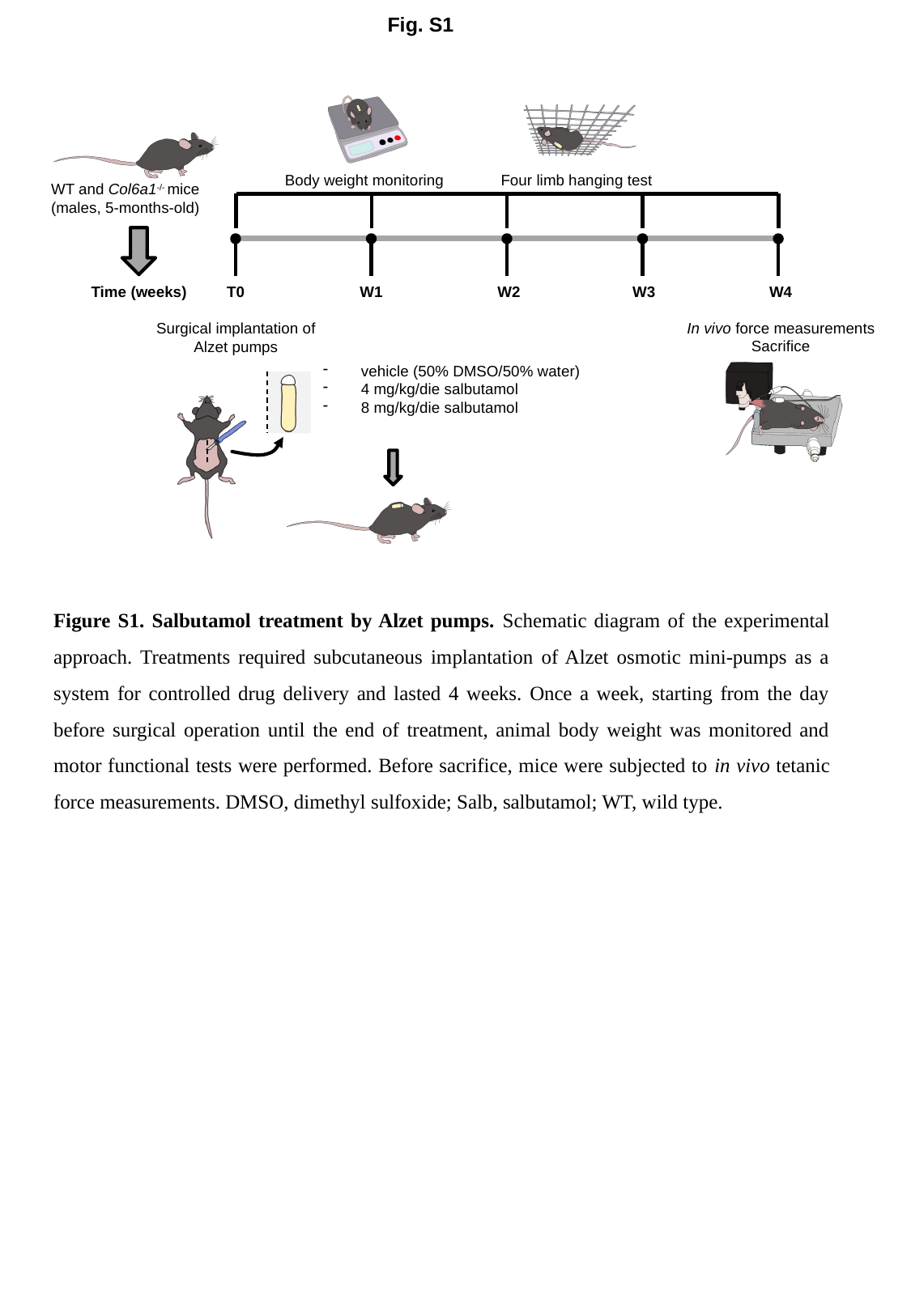

Fig. S1
Body weight monitoring
Four limb hanging test
WT and Col6a1-/- mice
(males, 5-months-old)
Time (weeks)
W1
W2
W3
W4
In vivo force measurements
Sacrifice
T0
Surgical implantation of Alzet pumps
vehicle (50% DMSO/50% water)
4 mg/kg/die salbutamol
8 mg/kg/die salbutamol
Figure S1. Salbutamol treatment by Alzet pumps. Schematic diagram of the experimental approach. Treatments required subcutaneous implantation of Alzet osmotic mini-pumps as a system for controlled drug delivery and lasted 4 weeks. Once a week, starting from the day before surgical operation until the end of treatment, animal body weight was monitored and motor functional tests were performed. Before sacrifice, mice were subjected to in vivo tetanic force measurements. DMSO, dimethyl sulfoxide; Salb, salbutamol; WT, wild type.

## Slide 2
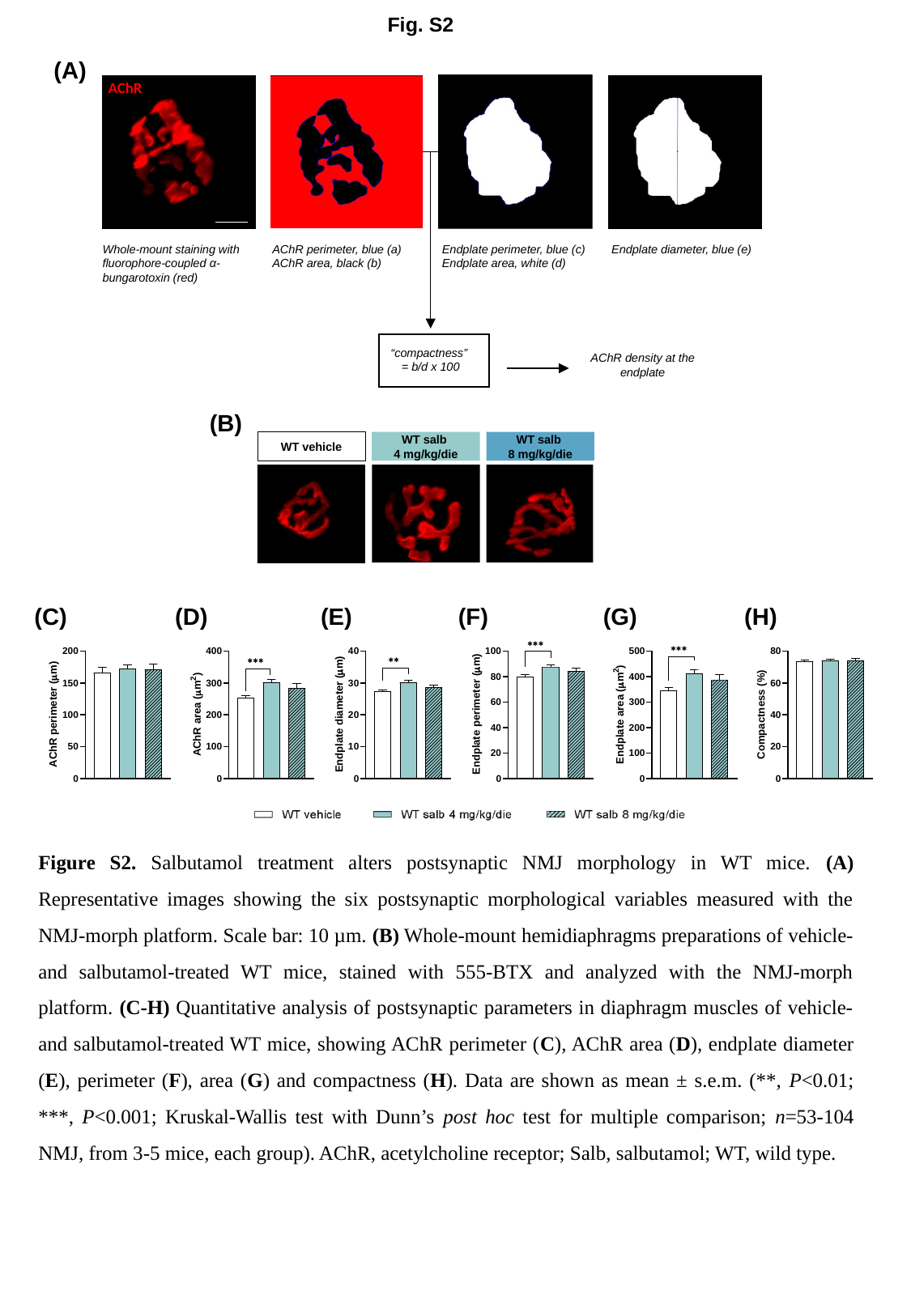

Fig. S2
(A)
AChR
AChR perimeter, blue (a)
AChR area, black (b)
Endplate perimeter, blue (c)
Endplate area, white (d)
Endplate diameter, blue (e)
“compactness”
= b/d x 100
Whole-mount staining with fluorophore-coupled α-bungarotoxin (red)
AChR density at the endplate
(B)
WT vehicle
WT salb
4 mg/kg/die
WT salb
8 mg/kg/die
10 μm
(C)
(D)
(E)
(F)
(G)
(H)
Figure S2. Salbutamol treatment alters postsynaptic NMJ morphology in WT mice. (A) Representative images showing the six postsynaptic morphological variables measured with the NMJ-morph platform. Scale bar: 10 µm. (B) Whole-mount hemidiaphragms preparations of vehicle- and salbutamol-treated WT mice, stained with 555-BTX and analyzed with the NMJ-morph platform. (C-H) Quantitative analysis of postsynaptic parameters in diaphragm muscles of vehicle- and salbutamol-treated WT mice, showing AChR perimeter (C), AChR area (D), endplate diameter (E), perimeter (F), area (G) and compactness (H). Data are shown as mean ± s.e.m. (**, P<0.01; ***, P<0.001; Kruskal-Wallis test with Dunn’s post hoc test for multiple comparison; n=53-104 NMJ, from 3-5 mice, each group). AChR, acetylcholine receptor; Salb, salbutamol; WT, wild type.

## Slide 3
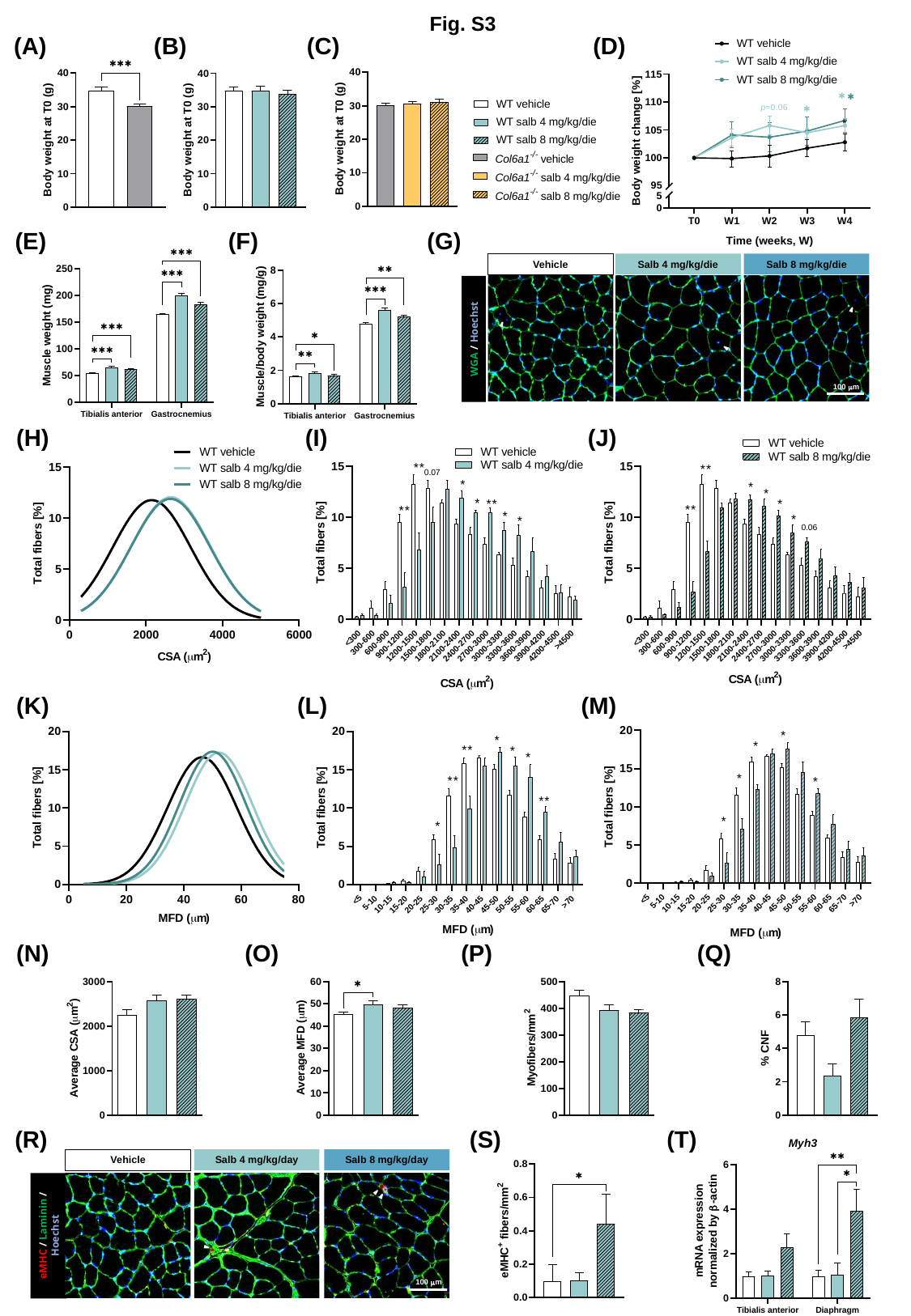

Fig. S3
(A)
(B)
(C)
(D)
(E)
(F)
(G)
Vehicle
Salb 4 mg/kg/die
Salb 8 mg/kg/die
WGA / Hoechst
100 μm
(H)
(I)
(J)
(K)
(L)
(M)
(N)
(O)
(P)
(Q)
(R)
(S)
(T)
Vehicle
Salb 4 mg/kg/day
Salb 8 mg/kg/day
eMHC / Laminin / Hoechst
100 μm

## Slide 4
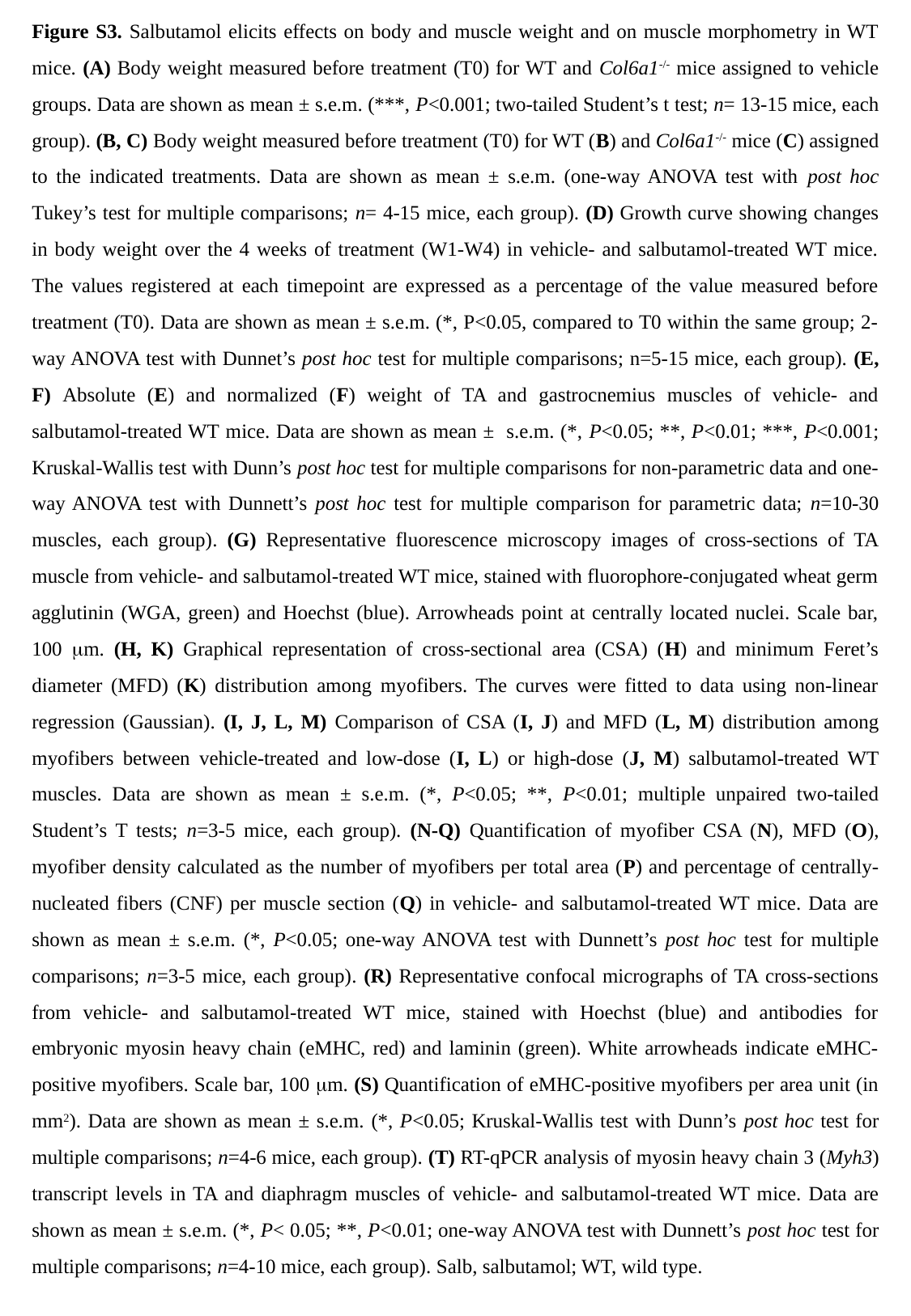

Figure S3. Salbutamol elicits effects on body and muscle weight and on muscle morphometry in WT mice. (A) Body weight measured before treatment (T0) for WT and Col6a1-/- mice assigned to vehicle groups. Data are shown as mean ± s.e.m. (***, P<0.001; two-tailed Student’s t test; n= 13-15 mice, each group). (B, C) Body weight measured before treatment (T0) for WT (B) and Col6a1-/- mice (C) assigned to the indicated treatments. Data are shown as mean ± s.e.m. (one-way ANOVA test with post hoc Tukey’s test for multiple comparisons; n= 4-15 mice, each group). (D) Growth curve showing changes in body weight over the 4 weeks of treatment (W1-W4) in vehicle- and salbutamol-treated WT mice. The values registered at each timepoint are expressed as a percentage of the value measured before treatment (T0). Data are shown as mean ± s.e.m. (*, P<0.05, compared to T0 within the same group; 2-way ANOVA test with Dunnet’s post hoc test for multiple comparisons; n=5-15 mice, each group). (E, F) Absolute (E) and normalized (F) weight of TA and gastrocnemius muscles of vehicle- and salbutamol-treated WT mice. Data are shown as mean ± s.e.m. (*, P<0.05; **, P<0.01; ***, P<0.001; Kruskal-Wallis test with Dunn’s post hoc test for multiple comparisons for non-parametric data and one-way ANOVA test with Dunnett’s post hoc test for multiple comparison for parametric data; n=10-30 muscles, each group). (G) Representative fluorescence microscopy images of cross-sections of TA muscle from vehicle- and salbutamol-treated WT mice, stained with fluorophore-conjugated wheat germ agglutinin (WGA, green) and Hoechst (blue). Arrowheads point at centrally located nuclei. Scale bar, 100 μm. (H, K) Graphical representation of cross-sectional area (CSA) (H) and minimum Feret’s diameter (MFD) (K) distribution among myofibers. The curves were fitted to data using non-linear regression (Gaussian). (I, J, L, M) Comparison of CSA (I, J) and MFD (L, M) distribution among myofibers between vehicle-treated and low-dose (I, L) or high-dose (J, M) salbutamol-treated WT muscles. Data are shown as mean ± s.e.m. (*, P<0.05; **, P<0.01; multiple unpaired two-tailed Student’s T tests; n=3-5 mice, each group). (N-Q) Quantification of myofiber CSA (N), MFD (O), myofiber density calculated as the number of myofibers per total area (P) and percentage of centrally-nucleated fibers (CNF) per muscle section (Q) in vehicle- and salbutamol-treated WT mice. Data are shown as mean ± s.e.m. (*, P<0.05; one-way ANOVA test with Dunnett’s post hoc test for multiple comparisons; n=3-5 mice, each group). (R) Representative confocal micrographs of TA cross-sections from vehicle- and salbutamol-treated WT mice, stained with Hoechst (blue) and antibodies for embryonic myosin heavy chain (eMHC, red) and laminin (green). White arrowheads indicate eMHC-positive myofibers. Scale bar, 100 μm. (S) Quantification of eMHC-positive myofibers per area unit (in mm2). Data are shown as mean ± s.e.m. (*, P<0.05; Kruskal-Wallis test with Dunn’s post hoc test for multiple comparisons; n=4-6 mice, each group). (T) RT-qPCR analysis of myosin heavy chain 3 (Myh3) transcript levels in TA and diaphragm muscles of vehicle- and salbutamol-treated WT mice. Data are shown as mean ± s.e.m. (*, P< 0.05; **, P<0.01; one-way ANOVA test with Dunnett’s post hoc test for multiple comparisons; n=4-10 mice, each group). Salb, salbutamol; WT, wild type.

## Slide 5
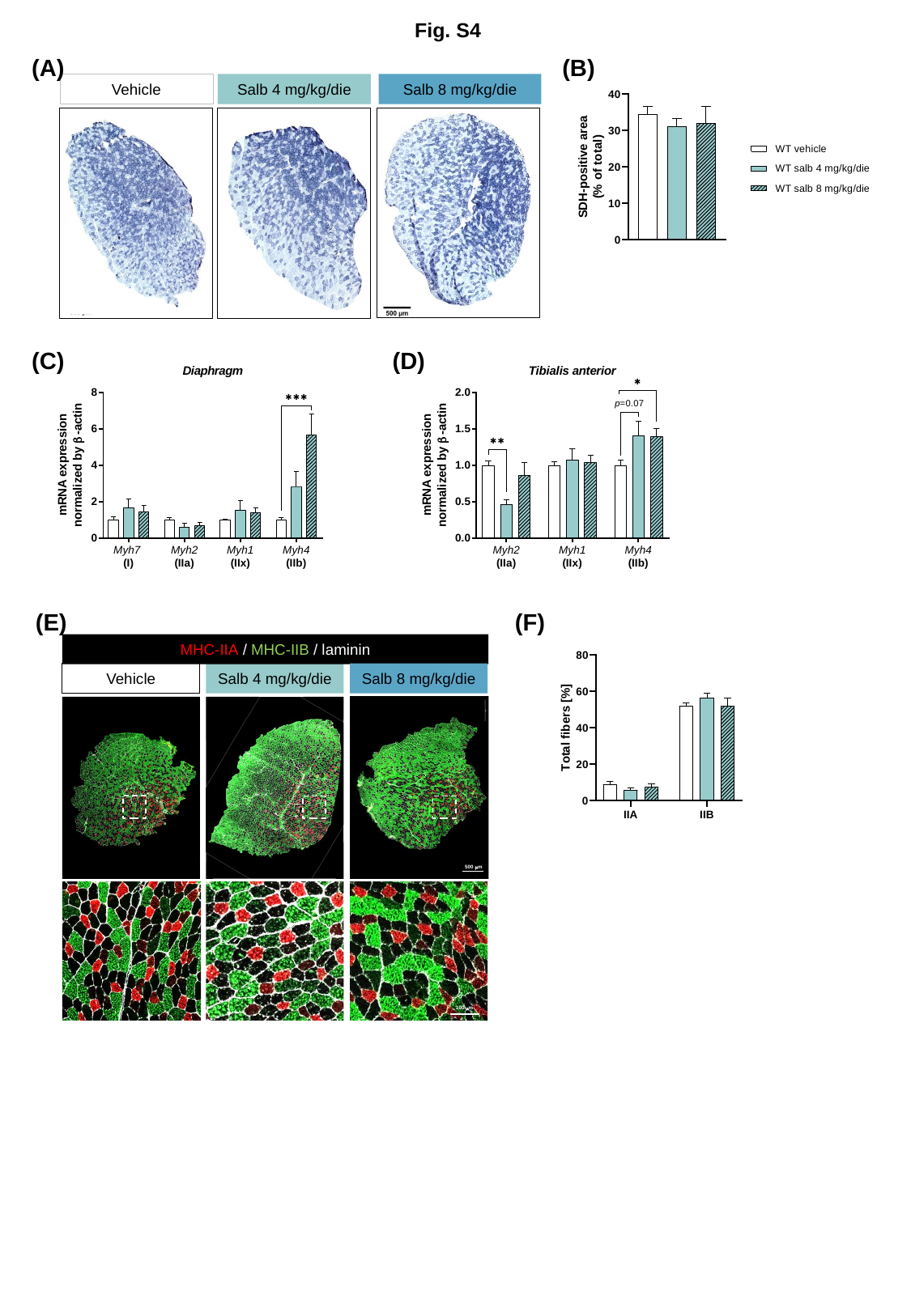

Fig. S4
(A)
(B)
Vehicle
Salb 4 mg/kg/die
Salb 8 mg/kg/die
(C)
(D)
(E)
(F)
MHC-IIA / MHC-IIB / laminin
Vehicle
Salb 4 mg/kg/die
Salb 8 mg/kg/die
500 μm
100 μm

## Slide 6
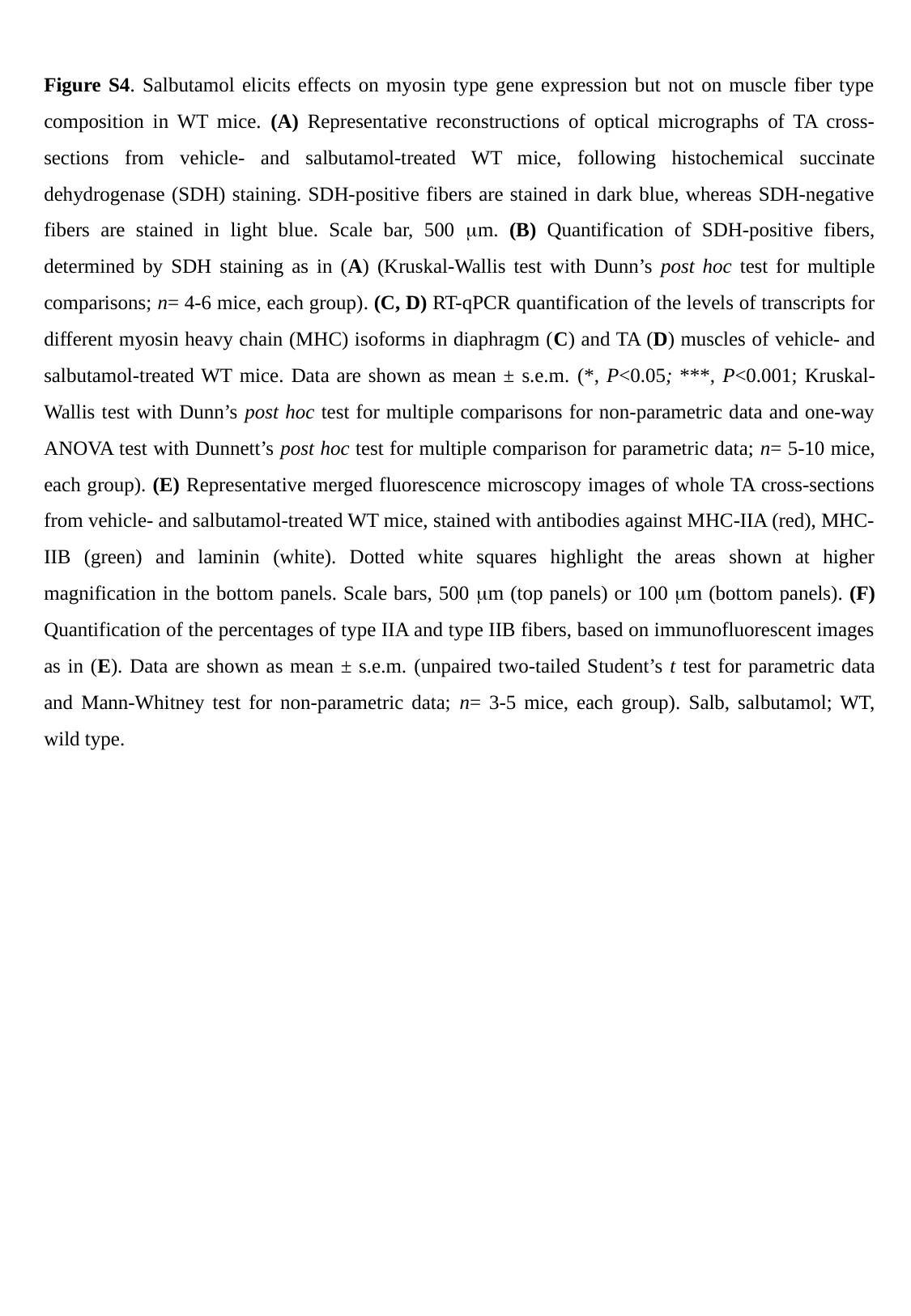

Figure S4. Salbutamol elicits effects on myosin type gene expression but not on muscle fiber type composition in WT mice. (A) Representative reconstructions of optical micrographs of TA cross-sections from vehicle- and salbutamol-treated WT mice, following histochemical succinate dehydrogenase (SDH) staining. SDH-positive fibers are stained in dark blue, whereas SDH-negative fibers are stained in light blue. Scale bar, 500 μm. (B) Quantification of SDH-positive fibers, determined by SDH staining as in (A) (Kruskal-Wallis test with Dunn’s post hoc test for multiple comparisons; n= 4-6 mice, each group). (C, D) RT-qPCR quantification of the levels of transcripts for different myosin heavy chain (MHC) isoforms in diaphragm (C) and TA (D) muscles of vehicle- and salbutamol-treated WT mice. Data are shown as mean ± s.e.m. (*, P<0.05; ***, P<0.001; Kruskal-Wallis test with Dunn’s post hoc test for multiple comparisons for non-parametric data and one-way ANOVA test with Dunnett’s post hoc test for multiple comparison for parametric data; n= 5-10 mice, each group). (E) Representative merged fluorescence microscopy images of whole TA cross-sections from vehicle- and salbutamol-treated WT mice, stained with antibodies against MHC-IIA (red), MHC-IIB (green) and laminin (white). Dotted white squares highlight the areas shown at higher magnification in the bottom panels. Scale bars, 500 μm (top panels) or 100 μm (bottom panels). (F) Quantification of the percentages of type IIA and type IIB fibers, based on immunofluorescent images as in (E). Data are shown as mean ± s.e.m. (unpaired two-tailed Student’s t test for parametric data and Mann-Whitney test for non-parametric data; n= 3-5 mice, each group). Salb, salbutamol; WT, wild type.

## Slide 7
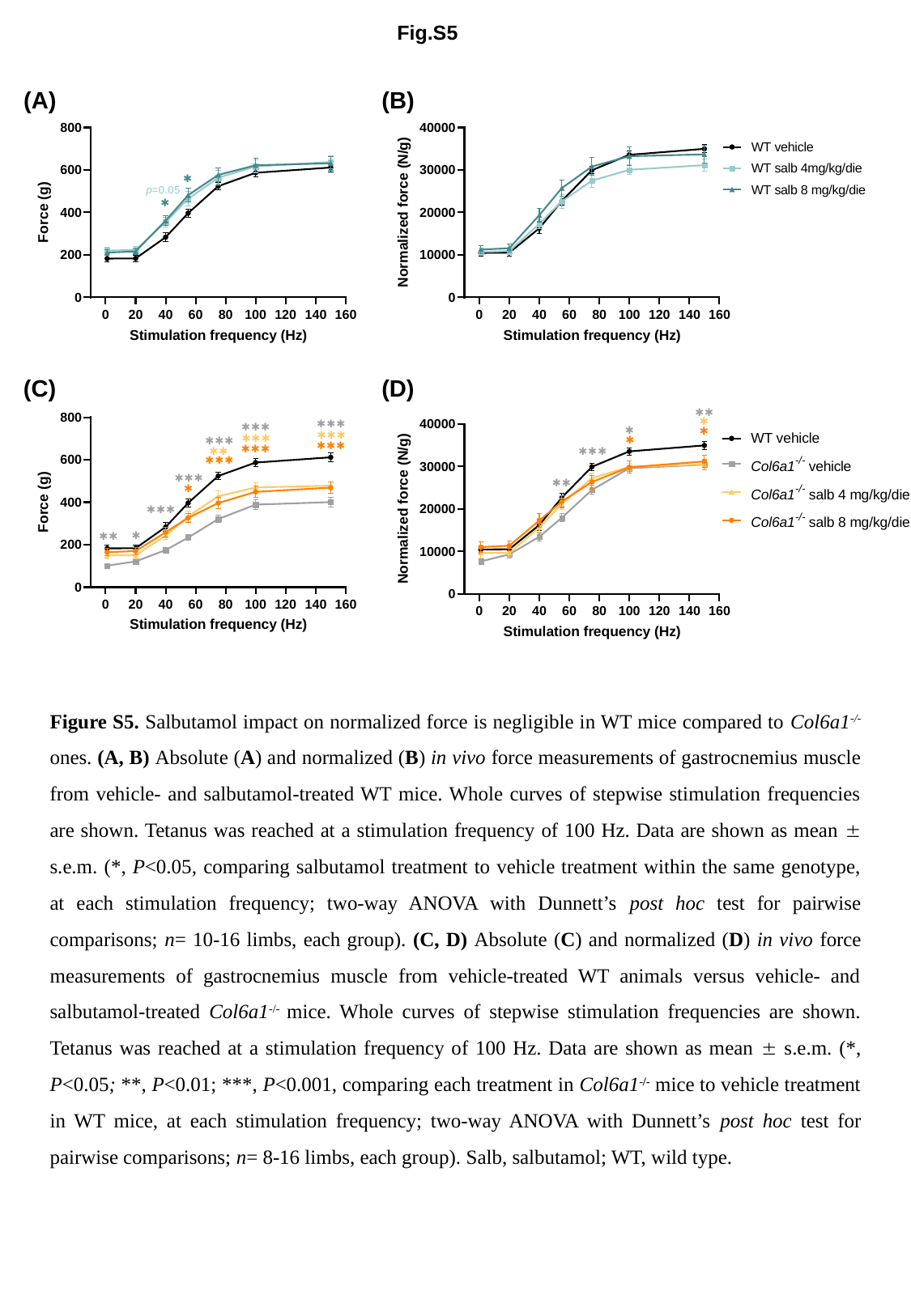

Fig.S5
(A)
(B)
(C)
(D)
Figure S5. Salbutamol impact on normalized force is negligible in WT mice compared to Col6a1-/- ones. (A, B) Absolute (A) and normalized (B) in vivo force measurements of gastrocnemius muscle from vehicle- and salbutamol-treated WT mice. Whole curves of stepwise stimulation frequencies are shown. Tetanus was reached at a stimulation frequency of 100 Hz. Data are shown as mean ± s.e.m. (*, P<0.05, comparing salbutamol treatment to vehicle treatment within the same genotype, at each stimulation frequency; two-way ANOVA with Dunnett’s post hoc test for pairwise comparisons; n= 10-16 limbs, each group). (C, D) Absolute (C) and normalized (D) in vivo force measurements of gastrocnemius muscle from vehicle-treated WT animals versus vehicle- and salbutamol-treated Col6a1-/- mice. Whole curves of stepwise stimulation frequencies are shown. Tetanus was reached at a stimulation frequency of 100 Hz. Data are shown as mean ± s.e.m. (*, P<0.05; **, P<0.01; ***, P<0.001, comparing each treatment in Col6a1-/- mice to vehicle treatment in WT mice, at each stimulation frequency; two-way ANOVA with Dunnett’s post hoc test for pairwise comparisons; n= 8-16 limbs, each group). Salb, salbutamol; WT, wild type.

## Slide 8
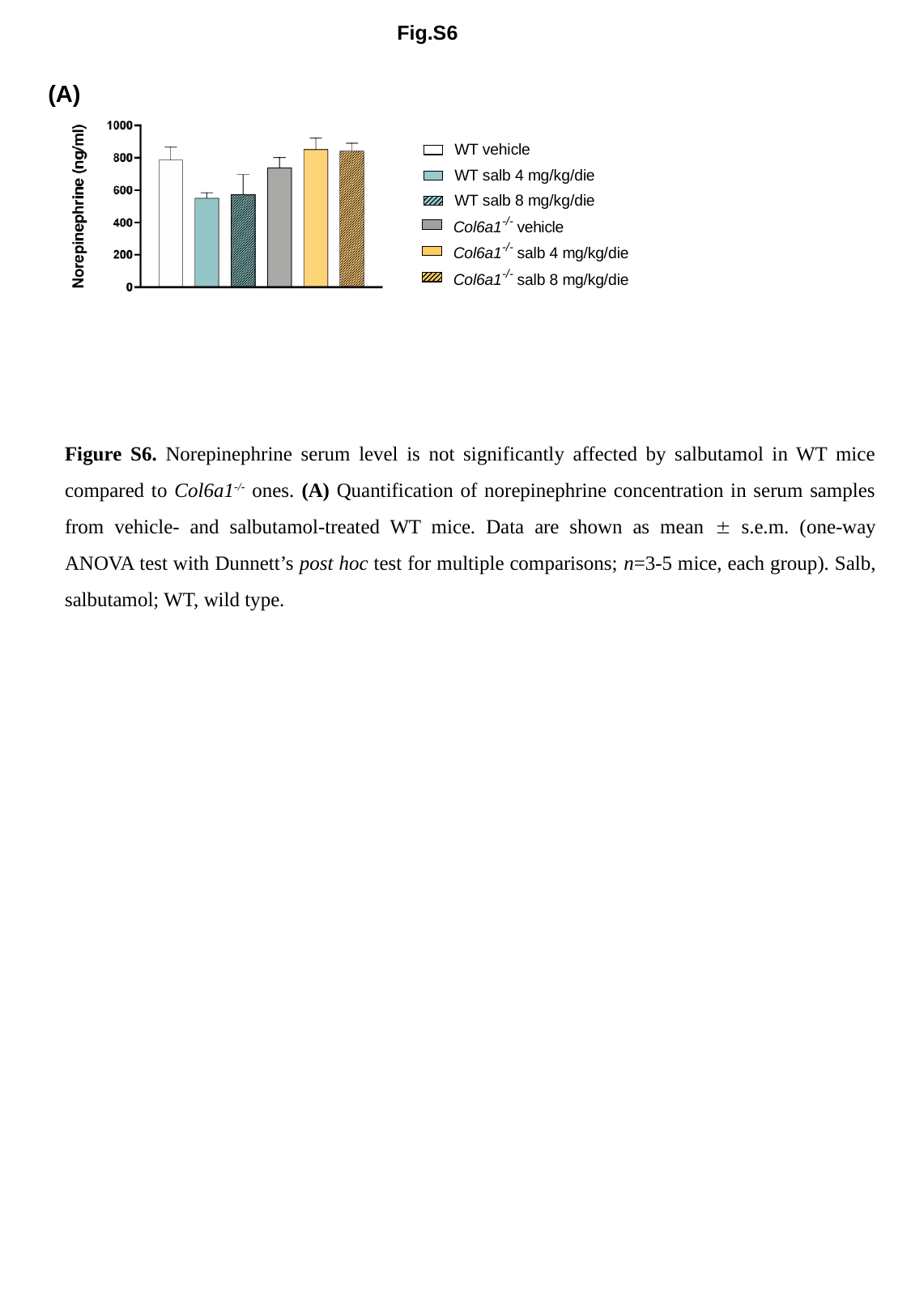

Fig.S6
(A)
Figure S6. Norepinephrine serum level is not significantly affected by salbutamol in WT mice compared to Col6a1-/- ones. (A) Quantification of norepinephrine concentration in serum samples from vehicle- and salbutamol-treated WT mice. Data are shown as mean ± s.e.m. (one-way ANOVA test with Dunnett’s post hoc test for multiple comparisons; n=3-5 mice, each group). Salb, salbutamol; WT, wild type.

## Slide 9
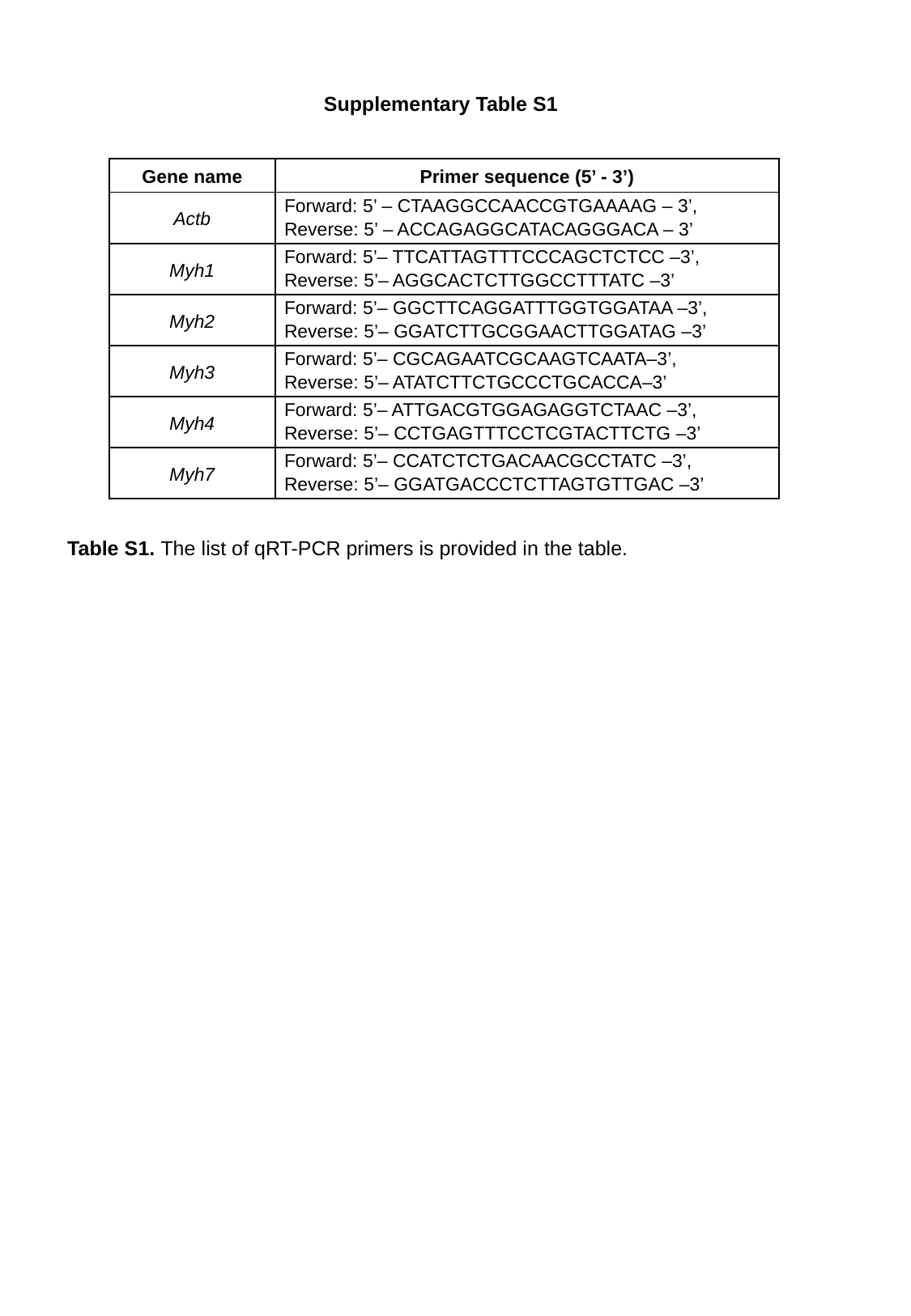

Supplementary Table S1
| Gene name | Primer sequence (5’ - 3’) |
| --- | --- |
| Actb | Forward: 5’ – CTAAGGCCAACCGTGAAAAG – 3’, Reverse: 5’ – ACCAGAGGCATACAGGGACA – 3’ |
| Myh1 | Forward: 5’– TTCATTAGTTTCCCAGCTCTCC –3’, Reverse: 5’– AGGCACTCTTGGCCTTTATC –3’ |
| Myh2 | Forward: 5’– GGCTTCAGGATTTGGTGGATAA –3’, Reverse: 5’– GGATCTTGCGGAACTTGGATAG –3’ |
| Myh3 | Forward: 5’– CGCAGAATCGCAAGTCAATA–3’, Reverse: 5’– ATATCTTCTGCCCTGCACCA–3’ |
| Myh4 | Forward: 5’– ATTGACGTGGAGAGGTCTAAC –3’, Reverse: 5’– CCTGAGTTTCCTCGTACTTCTG –3’ |
| Myh7 | Forward: 5’– CCATCTCTGACAACGCCTATC –3’, Reverse: 5’– GGATGACCCTCTTAGTGTTGAC –3’ |
Table S1. The list of qRT-PCR primers is provided in the table.
