# Supplementary figures and images for: Salbutamol repurposing ameliorates neuromuscular junction defects and muscle atrophy in Col6a1−/− mouse model of collagen VI‐related myopathies
Source: Clin Transl Med. 2024 Jul 10;14(7):e1688. doi: 10.1002/ctm2.1688 (PMC11234414; doi:10.1002/ctm2.1688)

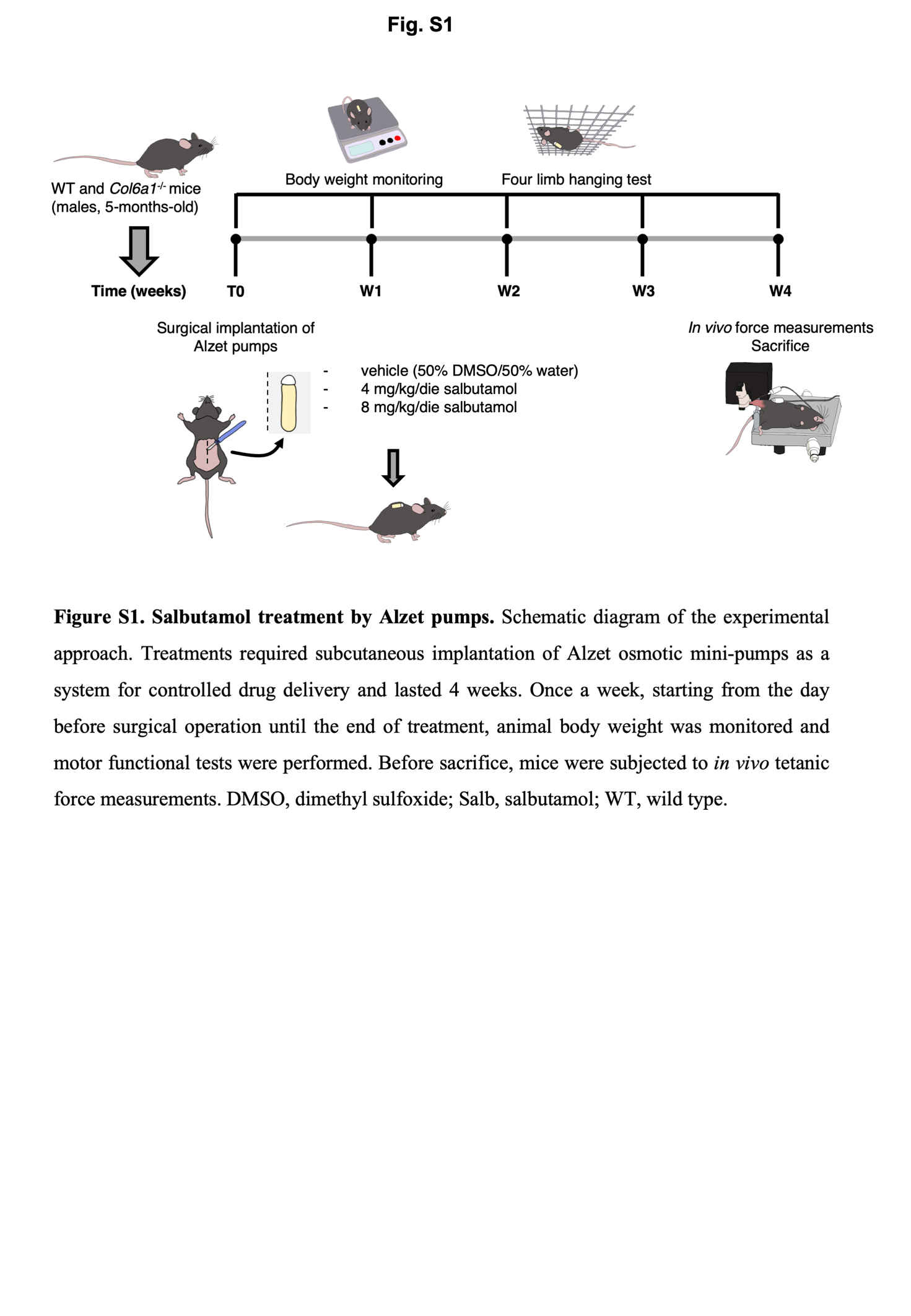


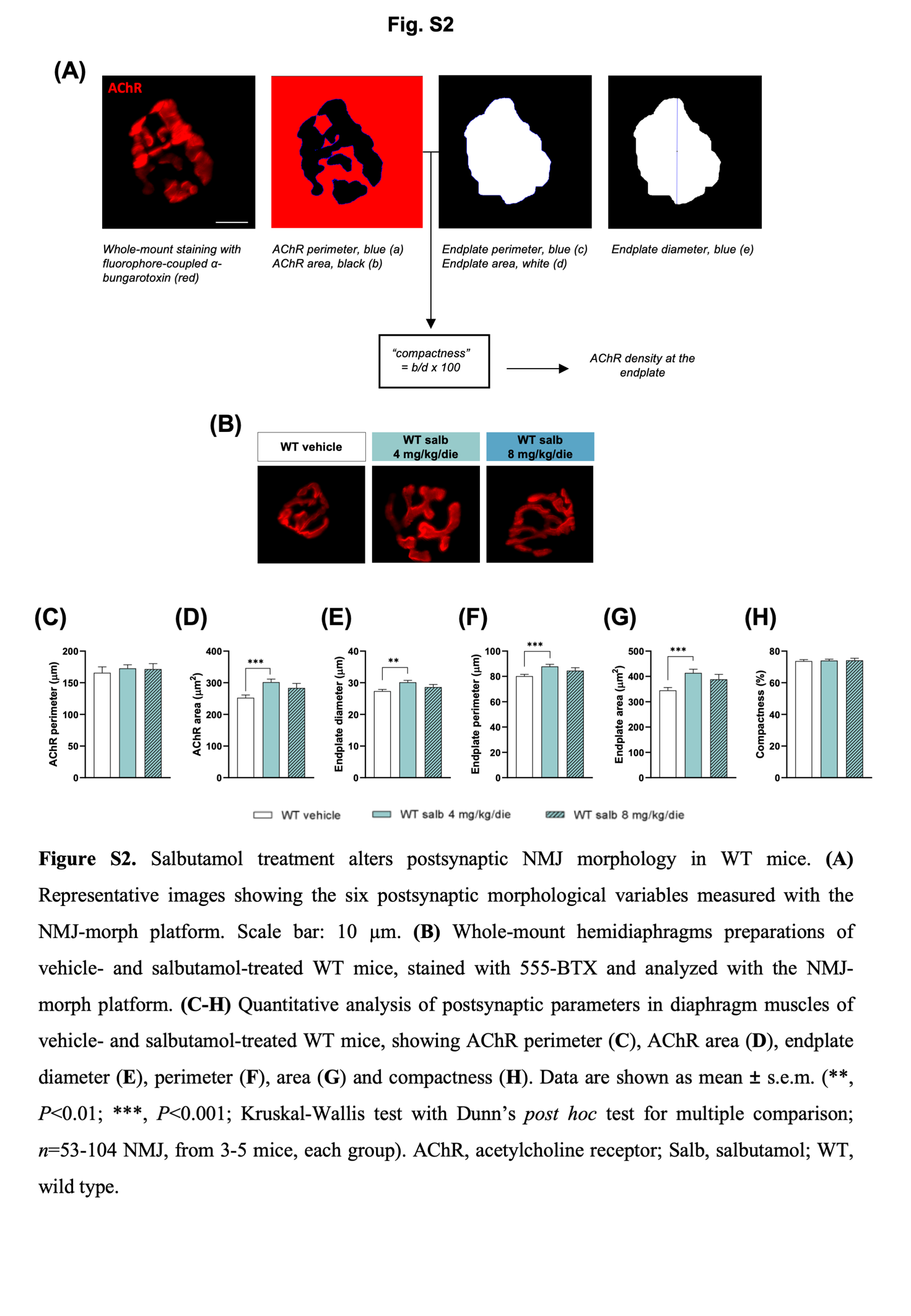


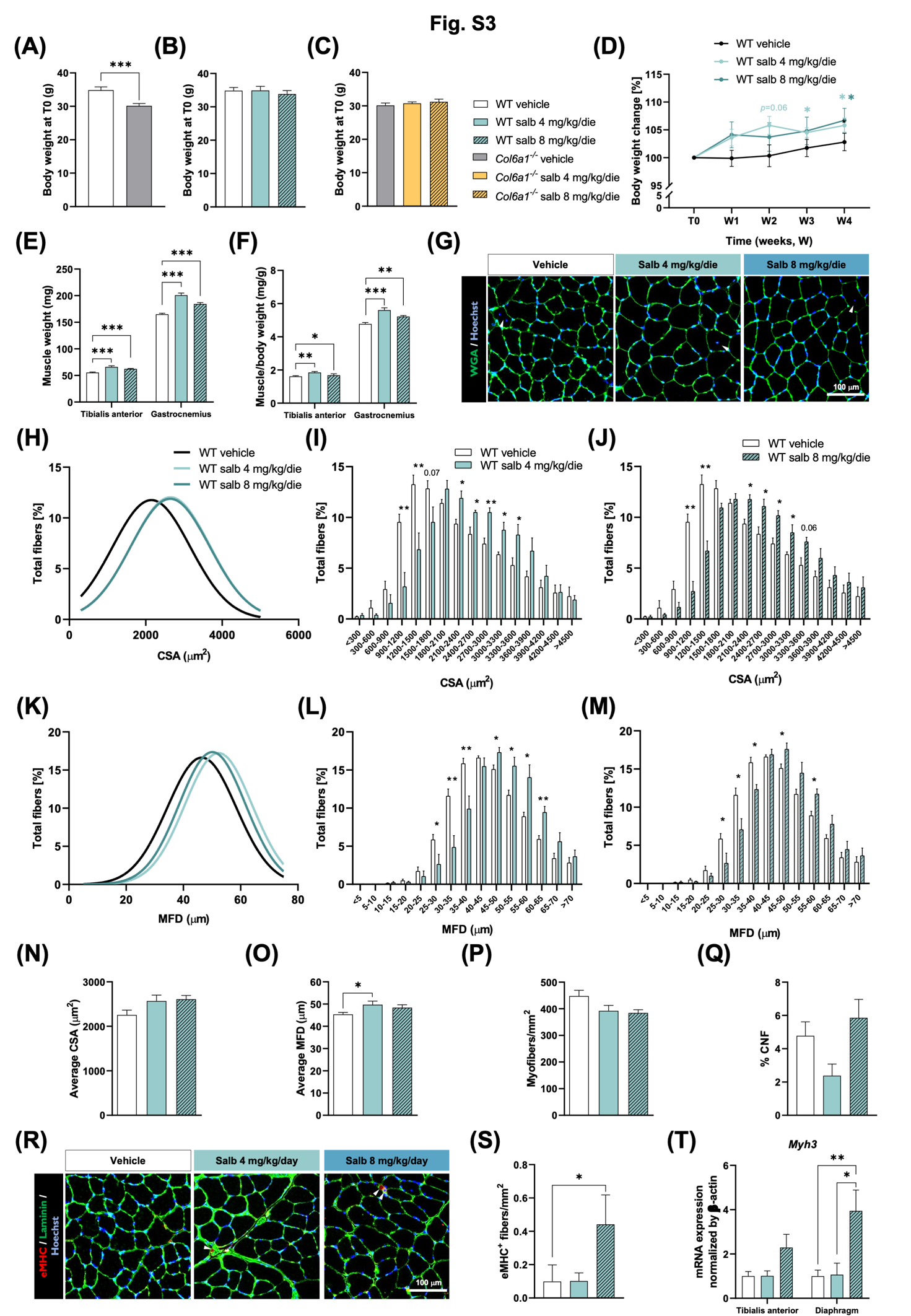


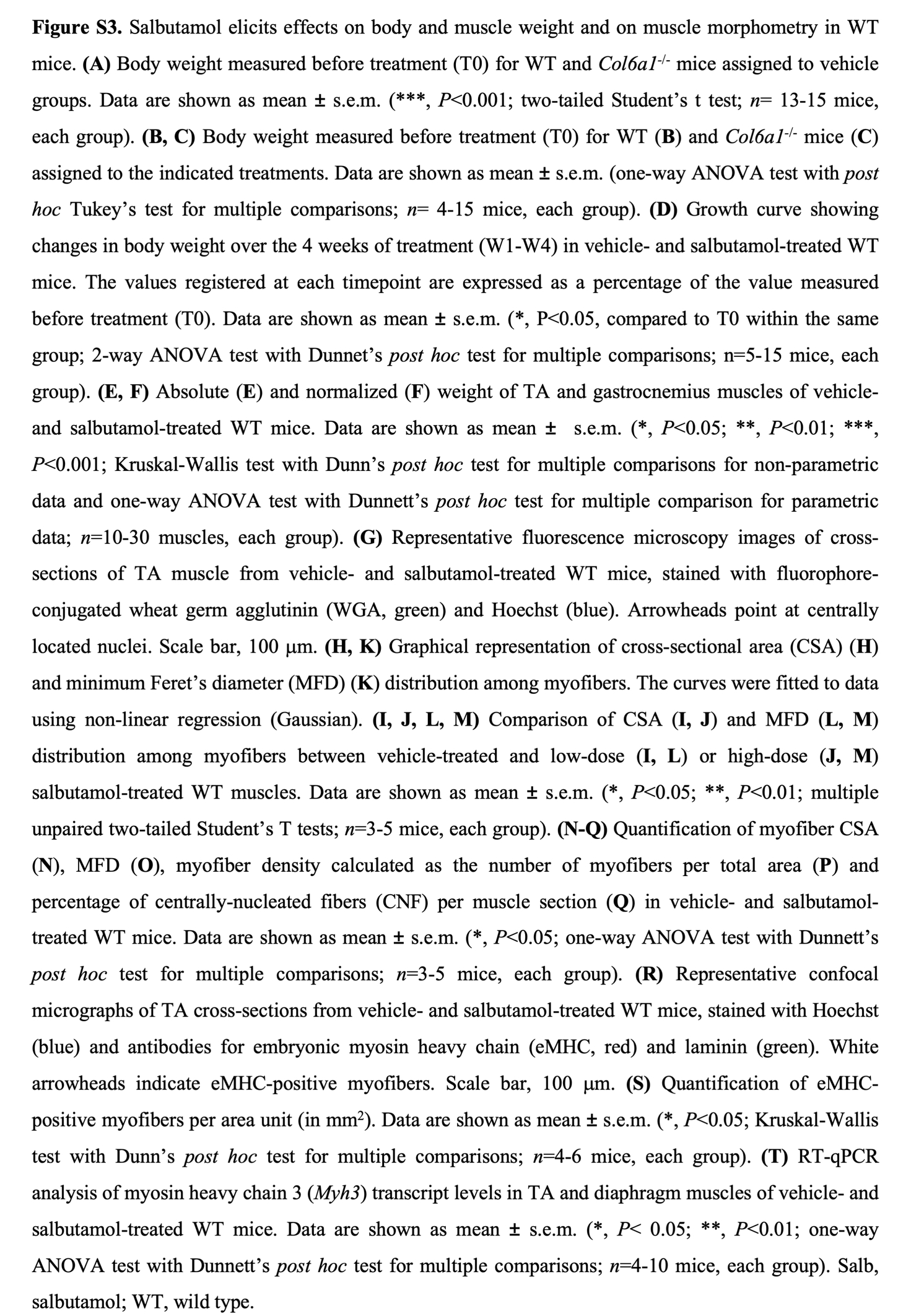


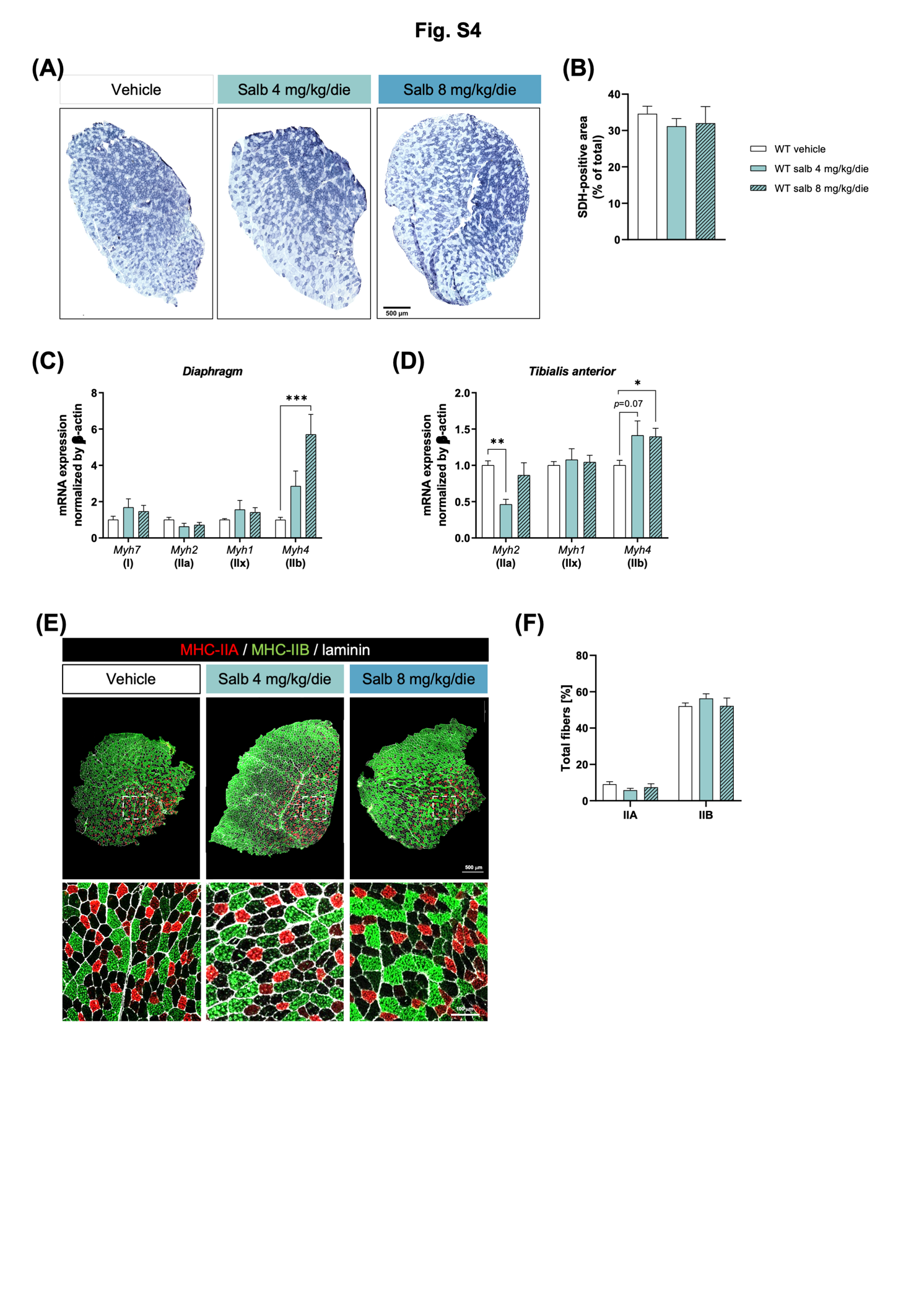


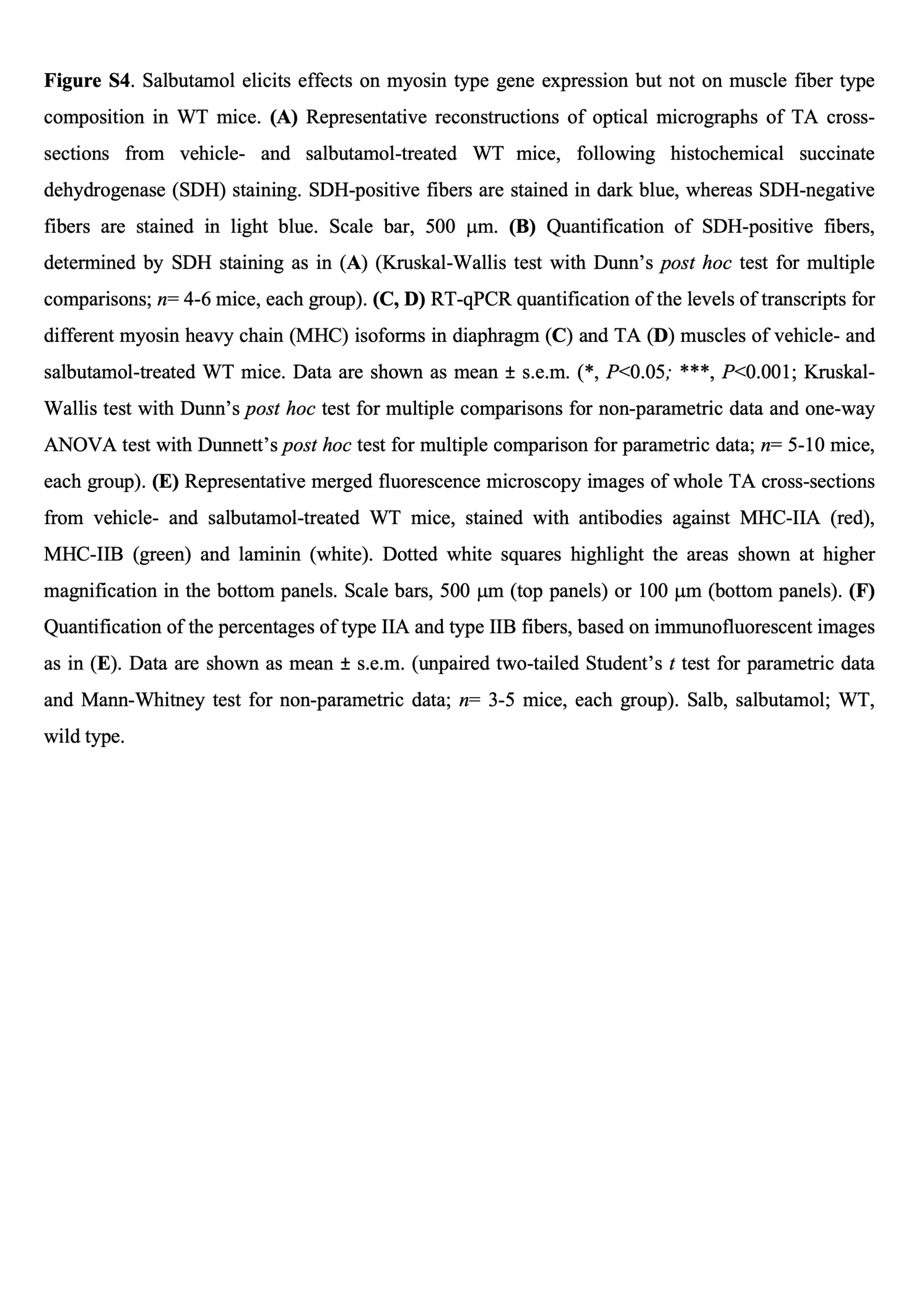


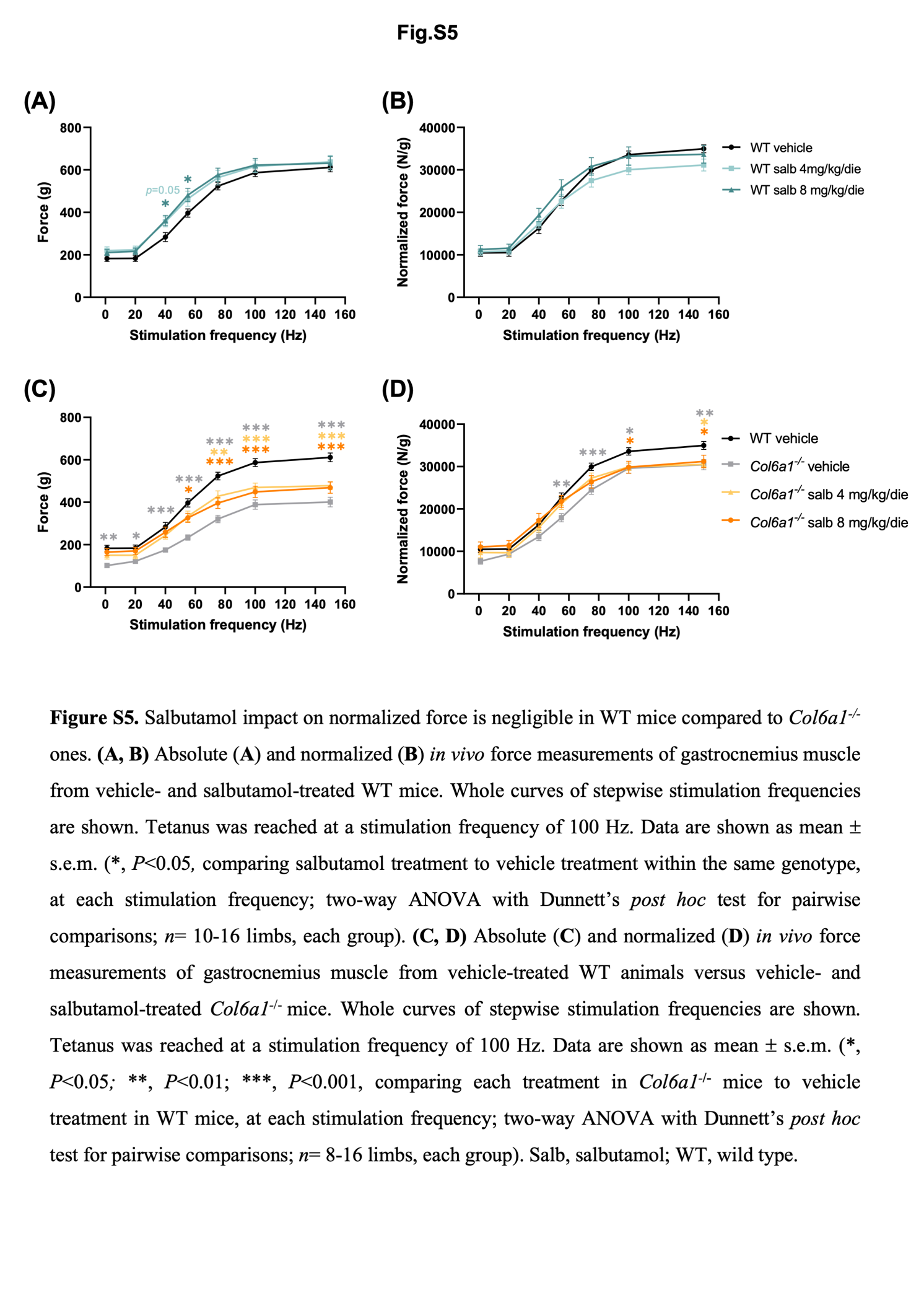


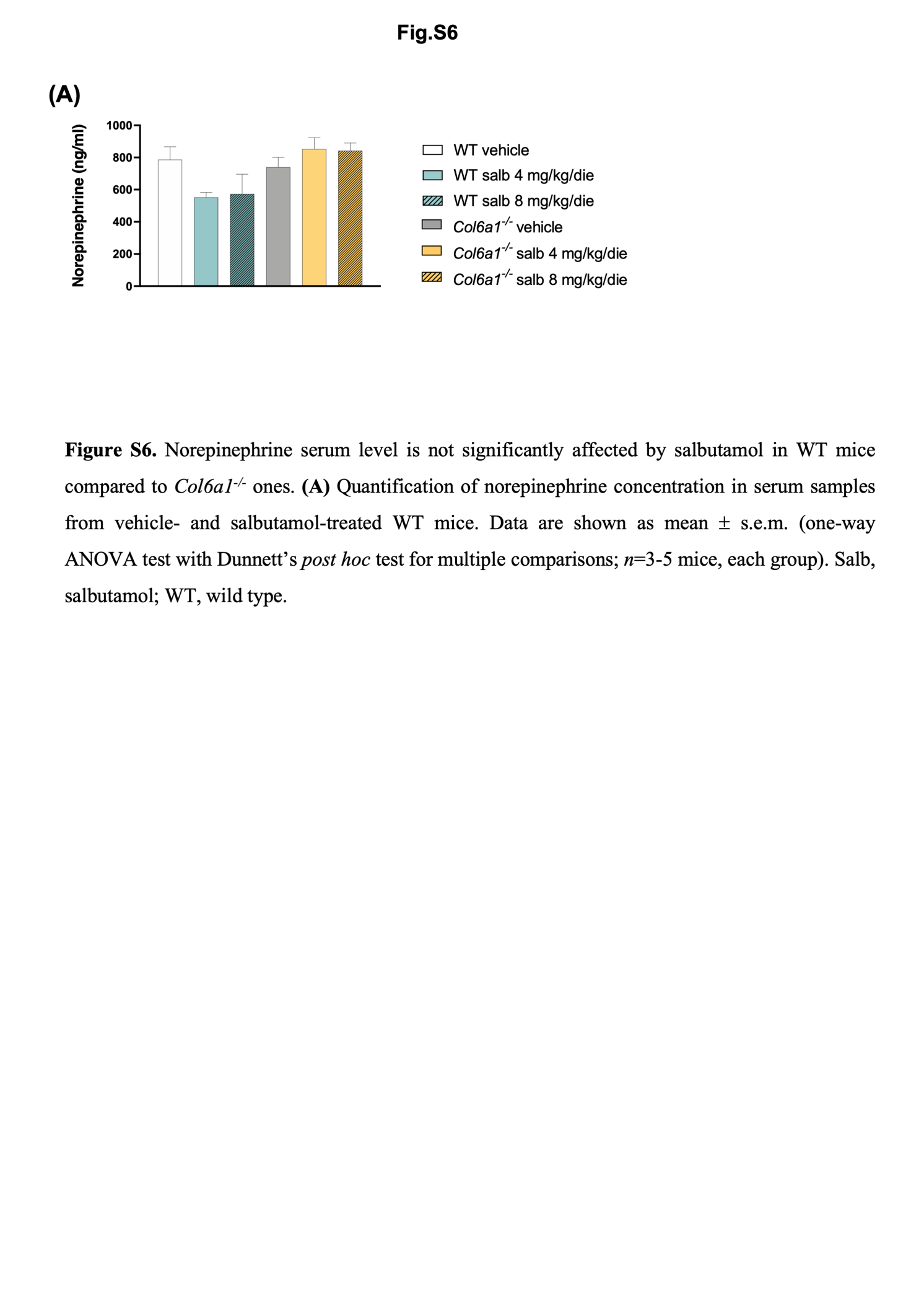


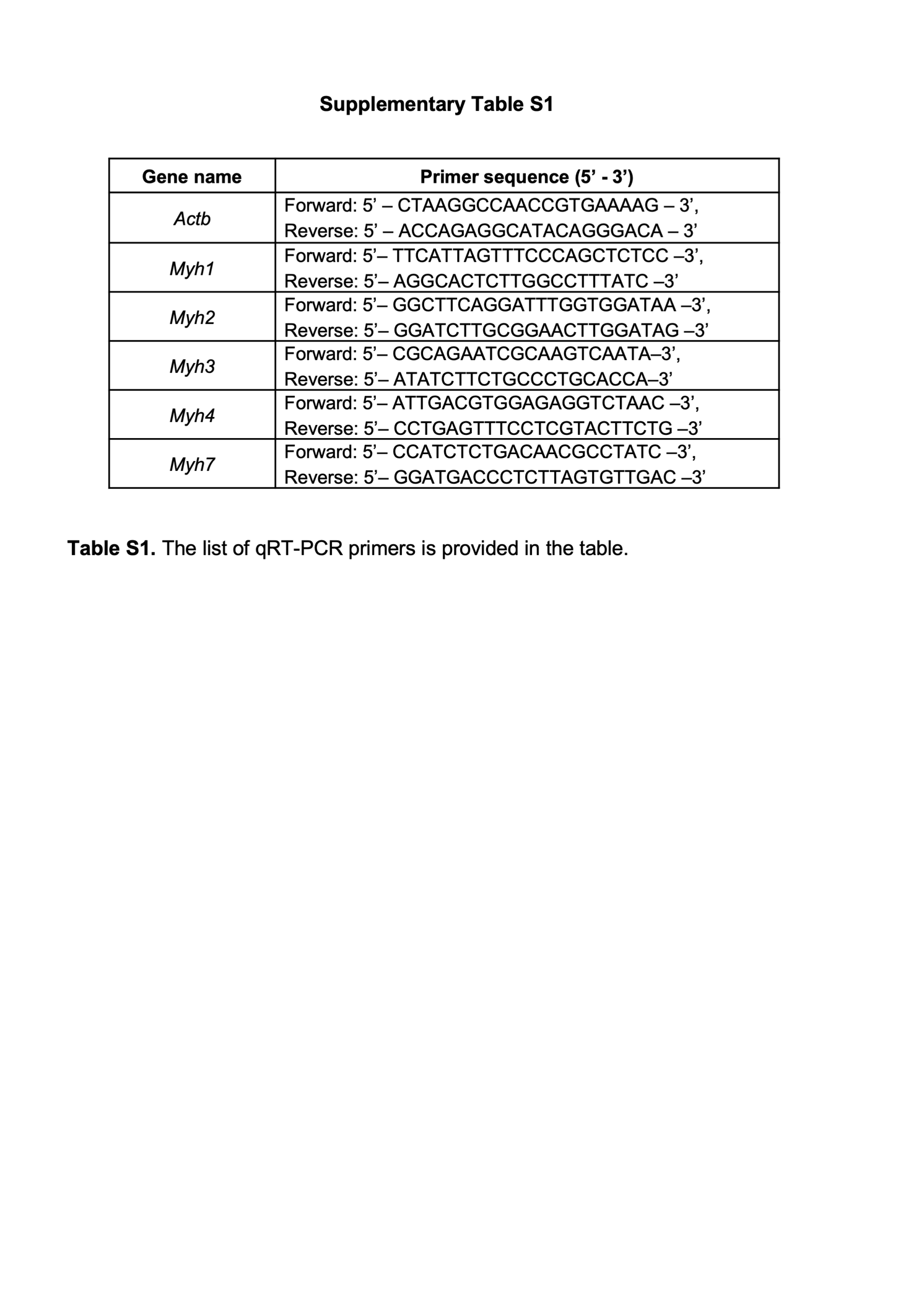

Supplement: Supplementary file 2 — Supporting Information [file CTM2-14-e1688-s001.docx]
